# Next‐generation sequencing reveals the mutational landscape of clinically diagnosed Usher syndrome: copy number variations, phenocopies, a predominant target for translational read‐through, and *PEX26* mutated in Heimler syndrome

**DOI:** 10.1002/mgg3.312

**Published:** 2017-07-06

**Authors:** Christine Neuhaus, Tobias Eisenberger, Christian Decker, Sandra Nagl, Cornelia Blank, Markus Pfister, Ingo Kennerknecht, Cornelie Müller‐Hofstede, Peter Charbel Issa, Raoul Heller, Bodo Beck, Klaus Rüther, Diana Mitter, Klaus Rohrschneider, Ute Steinhauer, Heike M. Korbmacher, Dagmar Huhle, Solaf M. Elsayed, Hesham M. Taha, Shahid M. Baig, Heidi Stöhr, Markus Preising, Susanne Markus, Fabian Moeller, Birgit Lorenz, Kerstin Nagel‐Wolfrum, Arif O. Khan, Hanno J. Bolz

**Affiliations:** ^1^ Bioscientia Center for Human Genetics Ingelheim Germany; ^2^ HNO‐Praxis Sarnen Sarnen Switzerland; ^3^ Molecular Genetics, THRC Department of Otolaryngology University of Tübingen Tübingen Germany; ^4^ Institute of Human Genetics Westfälische Wilhelms‐Universität Münster Germany; ^5^ Department of Ophthalmology University of Bonn Bonn Germany; ^6^ Center for Rare Diseases Bonn (ZSEB) University of Bonn Bonn Germany; ^7^ Oxford Eye Hospital University of Oxford Oxford UK; ^8^ Institute of Human Genetics University Hospital of Cologne Cologne Germany; ^9^ Sankt Gertrauden‐Krankenhaus Berlin Germany; ^10^ Institute of Human Genetics University of Leipzig Hospitals and Clinics Leipzig Germany; ^11^ Department of Ophthalmology University of Heidelberg Heidelberg Germany; ^12^ Cölbe Cölbe Germany; ^13^ Department of Orthodontics Giessen and Marburg University Hospital, Marburg Campus Marburg Germany; ^14^ Praxis für Humangenetik Leipzig Leipzig Germany; ^15^ Medical Genetics Center Cairo Egypt; ^16^ Children's Hospital Ain Shams University Cairo Egypt; ^17^ Human Molecular Genetics Laboratory Health Biotechnology Division National Institute for Biotechnology and Genetic Engineering (NIBGE) Faisalabad Pakistan; ^18^ Department of Human Genetics University Medical Center Regensburg Regensburg Germany; ^19^ Department of Ophthalmology Justus‐Liebig‐University Giessen Giessen Germany; ^20^ MVZ Dr. Staber und Kollegen GmbH Regensburg Germany; ^21^ Department of Cell and Matrix Biology Institute of Zoology, Johannes Gutenberg University of Mainz Mainz Germany; ^22^ Division of Pediatric Ophthalmology King Khaled Eye Specialist Hospital Riyadh Saudi Arabia; ^23^ Eye Institute Cleveland Clinic Abu Dhabi UAE

**Keywords:** Copy number variation, Heimler syndrome, next‐generation sequencing, phenocopies, translational read‐through, Usher syndrome

## Abstract

**Background:**

Combined retinal degeneration and sensorineural hearing impairment is mostly due to autosomal recessive Usher syndrome (USH1: congenital deafness, early retinitis pigmentosa (RP); USH2: progressive hearing impairment, RP).

**Methods:**

Sanger sequencing and NGS of 112 genes (Usher syndrome, nonsyndromic deafness, overlapping conditions), MLPA, and array‐CGH were conducted in 138 patients clinically diagnosed with Usher syndrome.

**Results:**

A molecular diagnosis was achieved in 97% of both USH1 and USH2 patients, with biallelic mutations in 97% (USH1) and 90% (USH2), respectively. Quantitative readout reliably detected CNVs (confirmed by MLPA or array‐CGH), qualifying targeted NGS as one tool for detecting point mutations and CNVs. CNVs accounted for 10% of identified *USH2A* alleles, often *in trans* to seemingly monoallelic point mutations. We demonstrate PTC124‐induced read‐through of the common p.Trp3955* nonsense mutation (13% of detected *USH2A* alleles), a potential therapy target. Usher gene mutations were found in most patients with atypical Usher syndrome, but the diagnosis was adjusted in case of double homozygosity for mutations in *OTOA* and *NR2E3*, genes implicated in isolated deafness and RP. Two patients with additional enamel dysplasia had biallelic *PEX26* mutations, for the first time linking this gene to Heimler syndrome.

**Conclusion:**

Targeted NGS not restricted to Usher genes proved beneficial in uncovering conditions mimicking Usher syndrome.

## Introduction

The co‐occurrence of bilateral hearing impairment (here comprehensively termed “deafness”) and visual impairment, if due to retinal degeneration, is of genetic origin in most cases in industrial countries. Usher syndrome mutations account for approximately 11% of deaf and hard of hearing children, and the population prevalence was estimated to be 1/6000 (Kimberling et al. 2010). The by far most prevalent causes are mutations in the 11 genes (*MYO7A*, OMIM *276903; *USH1C*, OMIM *605242; *CDH23*, OMIM *605516; *PCDH15*, OMIM *605514; *USH1G*, OMIM *607696; *CIB2*, OMIM *605564; *USH2A*, OMIM *608400; *ADGRV1*, OMIM *602851; *DFNB31*/*WHRN*, OMIM *607928; *CLRN1*, OMIM *606397; *PDZD7*, OMIM *612971) associated with Usher syndrome (Besnard et al. 2014), an autosomal recessive trait characterized by congenital deafness and RP in the first decade (in type 1, USH1; about 35% of cases (Petit 2001)) or by progressive hearing loss and RP of later onset in USH2 (about two thirds of patients). Symptoms apart from deaf‐blindness, however, may indicate other (genetic) diagnoses (e.g., disease related to mutations in *PEX1*, OMIM *602136, or *PEX6*, OMIM *601498). Especially in consanguineous families, simultaneous presence of two non‐syndromic sensory deficits must be taken into account. In our comprehensive analysis of a large cohort of deaf‐blindness patients clinically diagnosed as Usher syndrome, we therefore conducted both conventional Sanger and next‐generation sequencing (NGS) of a large gene panel not only comprising the Usher genes but also the known genes for non‐syndromic deafness and for syndromes that may comprise both sensory deficits. We efficiently established identification of CNVs from NGS data, highlighting targeted NGS as a tool for diagnosing both point mutations and copy number alterations. Simultaneous homozygosity for mutations in genes associated with isolated retinal degeneration and hearing loss (*OTOA*, OMIM *607038 and *NR2E3*, OMIM *604485), and mutations in *PEX26* (OMIM *608666) in patients with additional enamel dysplasia demonstrate how rare, genetically distinct entities may mimic Usher syndrome.

## Materials and Methods

### Ethical compliance

Samples were obtained with written informed consent. All investigations were conducted according to the Declaration of Helsinki, and the study was approved by the institutional review board of the Ethics Committee of the University Hospital of Cologne.

### Patients

**Figure 1:**
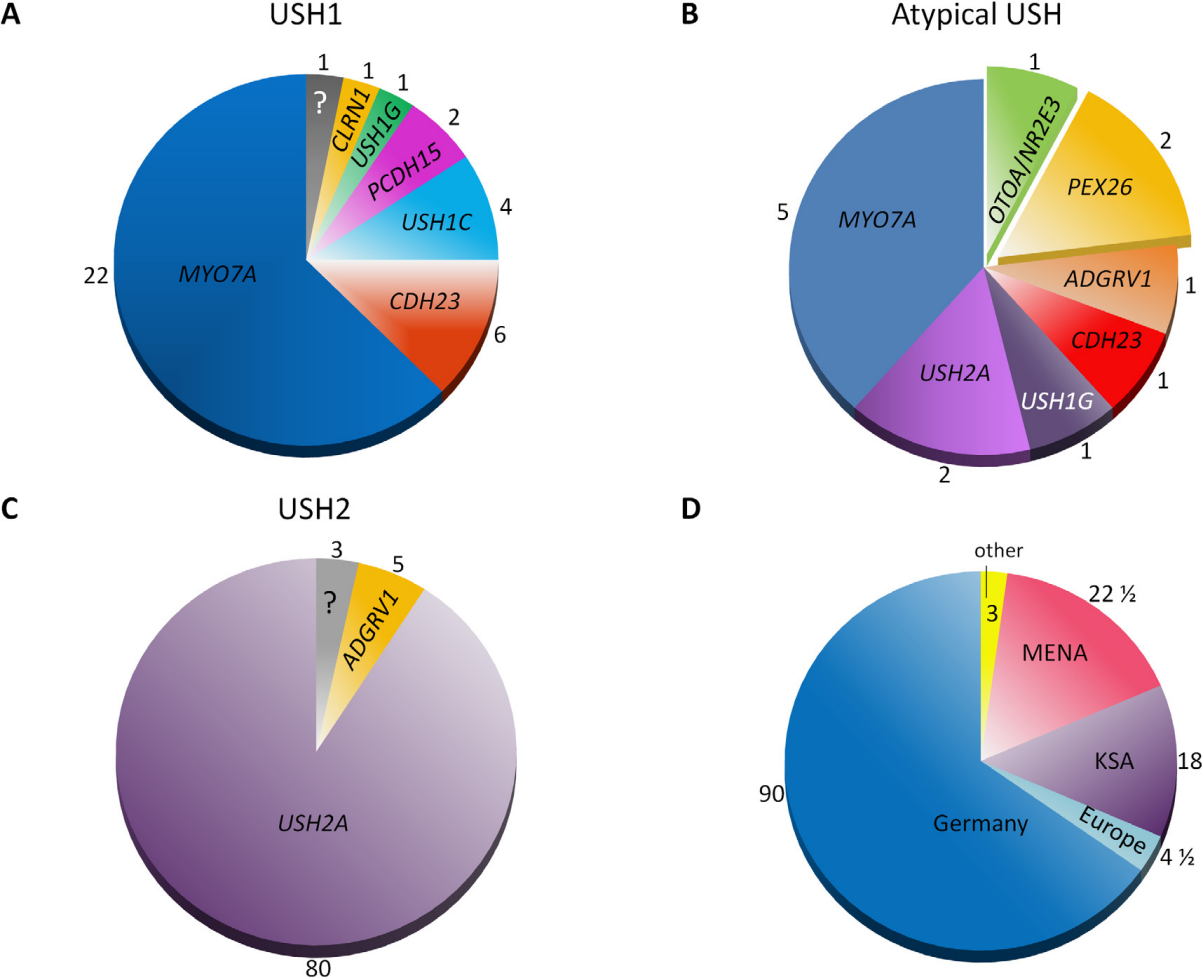
Diagnostic yield and mutational spectrum in patients clinically diagnosed with different types of Usher syndrome. Numbers correspond to patient numbers. ?, unsolved patients. (A) USH1. (B) Atypical Usher syndrome (including patients with additional, non‐sensory symptoms). (C) USH2. (D) Ethnic origin of patients. Patients were counted as ^1^/_2_ + ^1^/_2_ if parents had different ethnical backgrounds.

The patients had been referred to our diagnostic laboratory with the diagnosis of retinal degeneration and sensorineural hearing loss, and therefore in most cases with suspected Usher syndrome (see below for exceptions concerning deafness patients). About two third of the patients were of German descent, and the remaining one third were from Saudi Arabia (KSA) and other Middle East/North African (MENA) countries (Fig. 1D). Patients whose phenotype was compatible with USH1 or USH2 were grouped accordingly. Patients whose symptoms comprised retinal degeneration and hearing impairment but did neither correspond to USH1 nor to USH2 (either because of clinical course or “plus symptoms” that were unusual for Usher syndrome) were categorized as “atypical Usher syndrome”. In nine pediatric or adolescent patients with apparently non‐syndromic deafness who had been referred for genetic testing of hearing loss genes (including the most important syndrome genes like those for Usher syndrome), the diagnosis was reversed (to a syndrome with RP to develop in the future) due to the genetic findings. For clarity, and although these patients had not been referred as Usher syndrome patients, they were grouped retrospectively under the clinical subtype that is usually associated with the respective gene (Table 1).

**Table 1:** Mutations identified in our study

Pat	Gene	Allele 1		dbSNP	Ref	Met	Allele 2		dbSNP	S	Ref	Met	Age (years)	Cons	Origin
**USH1**															
101	*MYO7A*	c.397C>T	p.His133Tyr	rs111033403	Le Guedard‐Mereuze et al. (2010)	NGS	c.640G>A	p.Gly214Arg	rs111033283		Adato et al. (1997); Aparisi et al. (2013)	NGS	36	No	KSA
DEM289	*MYO7A*	c.397dupC	p.His133Profs*7	‐‐‐	nov	NGS	c.397dupC	p.His133Profs*7	‐‐‐		nov	NGS	29	Yes	Pak
DEM318	*MYO7A*	c.397dupC	p.His133Profs*7s	‐‐‐	nov	NGS	c.397dupC	p.His133Profs*7	‐‐‐		nov	NGS	n.d.	Yes	Pak
94	*MYO7A*	c.470+1G>A	Splice	‐‐‐	Adato et al. (1997)	NGS	c.470+1G>A	Splice	‐‐‐		Adato et al. (1997)	NGS	n.d.	Yes	KSA
102	*MYO7A*	c.470+1G>A	Splice	‐‐‐	Adato et al. (1997)	NGS	c.4818delG	p.Lys1606Asnfs*39	‐‐‐		‐‐‐	NGS	18	Yes	KSA
110	*MYO7A*	c.470+1G>A	Splice	‐‐‐	Adato et al. (1997)	NGS	c.470+1G>A	Splice	‐‐‐	x	Adato et al. (1997)	NGS	5	Yes	KSA
87	*MYO7A*	c.496delG	p.Glu166Argfs*5	rs111033448	Riazuddin et al. (2008)	SaS	c.5617C>T	p.Arg1873Trp	‐‐‐	x	Riazuddin et al. (2008)	SaS	35	No	Italy
DEM279	*MYO7A*	c.745G>T	p.Glu249*	‐‐‐	nov	NGS	c.745G>T	p.Glu249*	‐‐‐		nov	NGS	16	Yes	Pak
1	*MYO7A*	c.848T>C	p.Met283Thr	‐‐‐	nov	SaS	c.1093G>A	p.Asp365Asn	‐‐‐		nov	SaS	30	No	Turkey
63	*MYO7A*	c.1189G>A	p.Ala397Thr	‐‐‐	Kimberling et al. (2010)	NGS	Del ex21‐27	Truncation	‐‐‐	x	nov	NGS	29	No	Ger
88	*MYO7A*	c.1903T>C	p.Cys635Arg	‐‐‐	nov	NGS	c.1903T>C	p.Cys635Arg	‐‐‐		nov	NGS	11	Yes	KSA
53*	*MYO7A*	c.2181dupT	p.Leu728Serfs*6	‐‐‐	nov	NGS	c.5749G>T	p.Glu1917*	‐‐‐	x	Jacobson et al. (2009); Aparisi et al. (2014)	NGS	2	No	Ger
109	*MYO7A*	c.2904G>T	p.Glu968Asp	rs111033233	Bharadwaj et al. (2000)	SaS	c.6409G>A	p.Gly2137Arg	‐‐‐	x	nov	SaS	29	No	Ger/Per
130	*MYO7A*	c.2904G>T	p.Glu968Asp	rs111033233	Bharadwaj et al. (2000)	NGS	c.2904G>T	p.Glu968Asp	rs111033233		Bharadwaj et al. (2000)	NGS	28	Yes	Syria
104	*MYO7A*	c.3547C>A	p.Pro1183Thr	‐‐‐	Cremers et al. (2007)	NGS	c.5879_5880delAC (c.5637‐3C>G)	Truncation (Splice?)	‐‐‐ ‐‐‐	x	nov Cremers et al. (2007)	NGS	58	No	Ger
5	*MYO7A*	c.3719G>A	p.Arg1240Gln	rs111033178	Janecke et a(l. 1999); Jacobson et al. (2011)	NGS	c.5320T>G	p.Phe1774Va	rs62625014	x	Yoshimura et al. (2013)	NGS	9	No	Ger
50*	*MYO7A*	c.3719G>A	p.Arg1240Gln	rs111033178	Janecke et al. (1999); Jacobson et al. (2011)	NGS	c.3719G>A	p.Arg1240Gln	rs111033178	x	Janecke et al. (1999); Jacobson et al. (2011)	NGS	4	No	Ger
2	*MYO7A*	c.3747delG	p.Leu1249Leufs*14	‐‐‐	nov	SaS	c.3851_3878dup28	p.Leu1293Leufs*24	‐‐‐		nov	SaS	47	No	Turkey
99	*MYO7A*	c.3764delA	p.Lys1255Argfs*8	‐‐‐	Jaijo et al. (2007)	NGS	c.3764delA	p.Lys1255Argfs*8	‐‐‐	x	Jaijo et al. (2007)	NGS	34	No	Ger
90	*MYO7A*	c.5886_5888del	p.Phe1963del	rs111033232	Roux et al. (2006)	NGS	c.5886_5888del	p.Phe1963del	rs111033232		Roux et al. (2006)	NGS	37	Yes	KSA
9237*	*MYO7A*	c.5958dupA	p.Leu1987Thrfs*89		nov	NGS	c.5958dupA	p.Leu1987Thrfs*89			nov	NGS	9	Yes	Egypt
117	*MYO7A*	c.6231dupG	p.Lys2078Glufs*50	‐‐‐	nov	NGS	c.6231dupG	p.Lys2078Glufs*50	‐‐‐		nov	NGS	13	Yes	KSA
6	*CDH23*	c.1411_1412delins	p.Glu471Ser	‐‐‐	nov	NGS	c.3862C>T	p.Gln1288*	‐‐‐	x	nov	NGS	29	No	Sri L.
51*	*CDH23*	c.1528A>T	p.Lys510*	‐‐‐	nov	NGS	c.1528A>T	p.Lys510*	‐‐‐	x	nov	NGS	3 m	n.d.	KSA
DEM127	*CDH23*	c.1701_1702del	p.Gly568Cysfs*20	‐‐‐	nov	MS, SaS	c.1701_1702del	p.Gly568Cysfs*20	‐‐‐		nov	MS, SaS	n.d.	Yes	Pak
122	*CDH23*	c.4393dupG	p.Ala1465Glyfs*3	‐‐‐	nov	NGS	c.4393dupG	p.Ala1465Glyfs*3	‐‐‐	x	nov	NGS	15	n.d.	Syria
12	*CDH23*	c.6047‐9G>A	Splice	‐‐‐	von Brederlow et al. (2002)	NGS	c.6047‐9G>A	Splice	‐‐‐	x	von Brederlow et al. (2002)	NGS	13	No	Italy
DEM296	*CDH23*	c.6047‐9G>A	Splice	‐‐‐	von Brederlow et al. (2002)	NGS	c.6047‐9G>A	Splice	‐‐‐		von Brederlow et al. (2002)	NGS	n.d.	Yes	Pak
95	*CLRN1*	c.301_305del	p.Val101Serfs*27	‐‐‐	Akoury et al. (2011)	NGS	c.301_305del	p.Val101Serfs*27	‐‐‐		Akoury et al. (2011)	NGS	13	Yes	Leban
7	*PCDH15*	c.401G>A	p.Arg134Gln	rs137853003	Ahmed et al. (2003)	MS, SaS	Del ex1‐3	Truncation	‐‐‐	x	Aller et al. (2010a,b)	MS, MLPA	30	Yes	Syria/Turkey
8	*PCDH15*	Del ex1‐3	Truncation	‐‐‐	Aller et al. (2010a,b)	MS, MLPA	Del ex1‐3	Truncation	‐‐‐		Aller et al. (2010a,b)	MS, MLPA	n.d.	Yes	Syria
123	*USH1C*	c.521+1G>A	Splice	‐‐‐	nov	NGS	c.521+1G>A	Splice	‐‐‐		nov	NGS	18	Yes	Syria
96	*USH1C*	Del ex3‐27	Truncation	‐‐‐	Bitner‐Glindzicz et al. (2000)	NGS	Del ex3‐27	Truncation	‐‐‐		Bitner‐Glindzicz et al. (2000)	NGS	9	n.d.	KSA
97	*USH1C*	Del ex3‐27	Truncation	‐‐‐	Bitner‐Glindzicz et al. (2000)	CGH	Del ex3‐27	Truncation	‐‐‐		Bitner‐Glindzicz et al. (2000)	CGH	2	n.d.	KSA
9140*	*USH1C*	c.1210+1G>C	Splice	‐‐‐	nov	NGS	c.1210+1G>C	Splice	‐‐‐		nov	NGS	10	Yes	Egypt
134*	*USH1G*	c.1311delG	p.Lys438Argfs*6	‐‐‐	nov	NGS	c.1311delG	p.Lys438Argfs*6	‐‐‐		nov	NGS	9	Yes	KSA
**USH1**	**Unsolved**														
56	‐‐‐	‐‐‐	‐‐‐	‐‐‐	‐‐‐	SaS, NGS	‐‐‐	‐‐‐	‐‐‐		‐‐‐	SaS, NGS	34	No	Greece
**Atyp**															
103	*CDH23*	Dup ex19‐27	Truncation?	‐‐‐	nov	NGS	Dup ex19‐27	Truncation?	‐‐‐	x	nov	NGS	30	Yes	KSA
DEM74	*USH1G*	c.1373 A>T	p.Asp458Val	rs397517925	Kalay et al. (2005 )	GLA, SaS	c.1373 A>T	p.Asp458Val	rs397517925		Kalay et al. (2005 )	MS, SaS		Yes	Pak
30	*ADGRV1*	c.6981delT	p.Gly2328Valfs*7	‐‐‐	nov	NGS	c.14044‐1G>A	Splice	‐‐‐	x	nov	NGS			Ger
9	*MYO7A*	c.849+5G>A	Splice	‐‐‐	nov	NGS	c.3907_3910dup	p.Ala1304Aspfs*5	‐‐‐		nov	NGS	27	No	Ger
11	*MYO7A*	c.3262C>T	p.Gln1088*	‐‐‐	nov	NGS	c.6439‐2A>G	Splice	‐‐‐		Mutai et al. (2013); Glockle et al. (2014)	NGS	26	No	Ger
13	*MYO7A*	c.3503G>A	p.Arg1168Gln	‐‐‐	nov	NGS	c.6025delG	Truncation	‐‐‐		Bharadwaj et al. (2000)	NGS	48	No	Ger
14	*MYO7A*	c.3503G>A	p.Arg1168Gln	‐‐‐	Aparisi et al. (2014)	NGS	c.5573T>C	p.Leu1858Pro	‐‐‐		Bharadwaj et al. (2000)	NGS	44	No	Ger
10	*MYO7A*	c.3718C>T	p.Arg1240Trp	‐‐‐	Janecke et al. (1999); Cremers et al. (2007)	SaS	c.4814C>A	p.Ser1605Tyr	‐‐‐	x	nov	SaS	25	No	Ger
75	*USH2A*	c.1036A>C	p.Asn346His	‐‐‐	Weston et al. (2000)	NGS	c.7967delA	p.Asn2656Ilefs*18	‐‐‐	x	nov	NGS	22	No	Ger
136*	*PEX26*	c.3G>A	p.Met1?	‐‐‐	nov	NGS	c.292C>T	p.Arg98Trp	rs62641228		Matsumoto et al. (2003); Furuki et al. (2006); Berendse et al. (2016)	NGS	3	No	Ger
135	*PEX26*	c.127G>C	p.Asp43His	‐‐‐	nov	NGS	c.292C>T	p.Arg98Trp	rs62641228		Matsumoto et al. (2003); Furuki et al. (2006); Berendse et al. (2016)	NGS	13	No	Ger
93	*OTOA*	gene deletion	Gene loss	‐‐‐	Shahin et al. (2010); Sloan‐Heggen et al. (2016)	NGS	Gene deletion	Gene loss	‐‐‐		Shahin et al. (2010); Sloan‐Heggen et al. (2016)	NGS	6	Yes	KSA
	*NR2E3*	c.932G>A	p.Arg311Gln	‐‐‐	Haider et al. (2000)	SaS	c.932G>A	p.Arg311Gln	rs28937873		Haider et al. (2000)	SaS			
**Atyp**	**Monoallelic**														
57	*USH2A*	c.13316C>T	p.Thr4439Ile	‐‐‐	Dreyer et al. (2008)	NGS	‐‐‐	‐‐‐	‐‐‐		‐‐‐	NGS	86	No	Ger
**USH2**															
89	*USH2A*	c.486‐1G>C	Splice	‐‐‐	Cremers et al. (2007)	NGS	c.486‐1G>C	Splice	‐‐‐		Cremers et al. (2007)	NGS	17	Yes	KSA
118	*USH2A*	c.486‐1G>C	Splice	‐‐‐	Cremers et al. (2007)	NGS	c.486‐1G>C	Splice	‐‐‐		Cremers et al. (2007)	NGS	24	Yes	KSA
15	*USH2A*	c.486‐14G>A	Splice	‐‐‐	Le Guedard‐Mereuze et al. (2010); Neveling et al. (2012)	SaS	c.6805+1G>A	Splice	‐‐‐		nov	SaS	44	No	Ger
34	*USH2A*	c.653T>A	p.Val218Glu	‐‐‐	Leroy et al. (2001)	SaS	c.949C>A	Silent/Splice	‐‐‐		Pennings et al. (2004)	SaS	30	No	Ger
52	*USH2A*	c.653T>A	p.Val218Glu	‐‐‐	Leroy et al. (2001)	NGS	c.2276G>T	p.Cys759Phe	rs80338902	x	Rivolta et al. (2000); Garcia‐Garcia et al. (2011)	NGS	48	No	Ger
33	*USH2A*	c.653T>A	p.Val218Glu	‐‐‐	Leroy et al. (2001)	SaS	c.8681+1G>T	Splice	‐‐‐		nov	SaS	58	No	Ger
37	*USH2A*	c.775_776delAG	p.Ser259Phefs*63	‐‐‐	Seyedahmadi et al. (2004)	SaS	c.9424G>T	p.Gly3142*	‐‐‐	x	Baux et al. (2007)	SaS	40	No	Ger
125	*USH2A*	c.802G>A	p.Gly268Arg	‐‐‐	Dreyer et al. (2008); Huang et al. (2015)	SaS	c.9346C>A	p.Pro3116Thr	‐‐‐	x	nov	SaS	44	No	Ger
41	*USH2A*	c.920_923dup	p.His308Glnfs*16	‐‐‐	Weston et al. (2000)	SaS	Del ex38‐41	Truncation	‐‐‐		nov	MLPA	26	No	Ger
132	*USH2A*	c.920_923dup	p.His308Glnfs*16	‐‐‐	Weston et al. (2000)	NGS	c.6084T>A	p.Tyr2028*	‐‐‐		Krawitz et al. (2014)	NGS	44	No	Ger
43	*USH2A*	c.949C>A	Silent/Splice	‐‐‐	Pennings et al. (2004)	SaS	c.14131C>T	p.Gln4711*	‐‐‐		McGee et al. (2010)	SaS	48	No	Ger
58	*USH2A*	c.1000C>T	p.Arg334Trp	‐‐‐	Adato et al. (2000)	NGS	c.6805+2T>C	Splice	‐‐‐	x	Krawitz et al. (2014)	NGS	60	No	Ger
121	*USH2A*	c.1036A>C	p.Asn346His	rs369522997	Weston et al. (2000); Sadeghi et al. (2013); Wang et al. (2014); Lenassi et al. (2015a,b)	SaS	c.8723_8724del	p.Val2908Glyfs*29	‐‐‐	x	van Wijk et al. (2004)	SaS	32	No	Ger
73	*USH2A*	c.1036A>C	p.Asn346His	rs369522997	Weston et al. (2000); Sadeghi et al. (2013); Wang et al. (2014); Lenassi et al. (2015a,b)	SaS	c.10561T>C	p.Trp3521Arg	rs111033264	x	Dreyer et al. (2008); McGee et al. (2010)	SaS	60	No	Ger
42	*USH2A*	c.1039G>C	p.Asp347His	‐‐‐	Pierrache et al. (2016)	SaS	c.14131C>T	p.Gln4711*	‐‐‐		McGee et al. (2010)	SaS	38	No	Ger
76	*USH2A*	c.1606T>C	p.Cys536Arg	rs111033273	Dreyer et al. (2000; Bhattacharya et al. (2004)	NGS	c.1606T>C	p.Cys536Arg	rs111033273		Dreyer et al. (2000); Bhattacharya et al. (2004)	NGS	19	No	Ger
68	*USH2A*	c.1752C>A	p.Cys584*	‐‐‐	nov	SaS	c.5122G>A	p.Gly1708Arg	‐‐‐		nov	SaS	45	No	Ger
131	*USH2A*	c.1876C>T	p.Arg626*	rs534534437	Weston et al. (2000)	NGS	c.11864G>A	p.Trp3955*	rs111033364		van Wijk et al. (2004); Le Quesne Stabej et al. (2012)	NGS	36	No	Ger
8	*USH2A*	c.2073C>A	p.Cys691*	‐‐‐	Seyedahmadi et al. (2004)	NGS	c.2209C>T	p.Arg737*	rs111033334		Kaiserman et al. (2007)	NGS	76	No	Ger
100	*USH2A*	c.2209C>T	p.Arg737*	rs111033334	Kaiserman et al. (2007)	NGS	c.6657+3_6657+6del	Splice	‐‐‐		nov	NGS	23	No	Ger
28	*USH2A*	c.2299delG	p.Glu767Serfs*21	rs80338903	Eudy et al. (1998)	SaS	c.8682‐9A>G	Splice	‐‐‐	x	Dreyer et al. (2008); Glockle et al. (2014)	SaS	59	No	Ger
32	*USH2A*	c.2299delG	p.Glu767Serfs*21	rs80338903	Eudy et al. (1998)	SaS	c.920_923dup	p.His308Glnfs*16	‐‐‐		Weston et al. (2000)	SaS	69	No	Ger
46	*USH2A*	c.2299delG	p.Glu767Serfs*21	rs80338903	Eudy et al. (1998)	SaS	c.949C>A	Silent/Splice	‐‐‐		Pennings et al. (2004)	SaS	47	No	Ger
31	*USH2A*	c.2299delG	p.Glu767Serfs*21	rs80338903	Eudy et al. 1998)	SaS	c.2299delG	p.Glu767Serfs*21	rs80338903	x	Eudy et al. (1998)	SaS	35	No	Ger
59	*USH2A*	c.2299delG	p.Glu767Serfs*21	rs80338903	Eudy et al. (1998)	SaS	c.2299delG	p.Glu767Serfs*21	rs80338903		Eudy et al. (1998)	SaS	25	No	Ger
47	*USH2A*	c.2299delG	p.Glu767Serfs*21	rs80338903	Eudy et al. (1998)	SaS	c.2610C>A	p.Cys870*	‐‐‐	x	Le Quesne Stabej et al. (2012)	SaS	25	No	Ger/Bos
127	*USH2A*	c.2299delG	p.Glu767Serfs*21	rs80338903	Eudy et al. (1998)	SaS	c.4714C>T (c.5516T>A)	p.Leu1572Phe (p.Val1839Glu)	rs111033333 ‐‐‐		Song et al. (2011); Zhao et al. (2015); Sloan‐Heggen et al. (2016) nov	SaS	33	No	Ger
108	*USH2A*	c.2299delG	p.Glu767Serfs*21	rs80338903	Eudy et al. (1998)	SaS	c.8522G>A	p.Trp2841*	‐‐‐		Sloan‐Heggen et al. (2016)	SaS	30	No	Ger
72	*USH2A*	c.2299delG	p.Glu767Serfs*21	rs80338903	Eudy et al. (1998)	SaS	c.11831C>A	p.Ala3944Asp	‐‐‐		Krawitz et al. (2014)	SaS	26	No	Ger
79*	*USH2A*	c.2299delG	p.Glu767Serfs*21	rs80338903	Eudy et al. (1998)	NGS	c.11864G>A	p.Trp3955*	rs111033364	x	van Wijk et al. (2004); Le Quesne Stabej et al. (2012)	NGS	15	No	Ger
44	*USH2A*	c.2299delG	p.Glu767Serfs*21	rs80338903	Eudy et al. (1998)	SaS	Del ex22‐24	Truncation	‐‐‐		Krawitz et al. (2014); Dad et al. (2015)	MLPA	43	No	Ger
78*	*USH2A*	c.2299delG	p.Glu767Serfs*21	rs80338903	Eudy et al. (1998)	NGS	Del ex15‐21	Truncation	‐‐‐	X	Baux et al. (2007)	NGS	1	No	Ger
71	*USH2A*	c.3648C>A	p.Tyr1216*	‐‐‐	nov	SaS	Del ex22‐24	Truncation	‐‐‐	x	Krawitz et al. (2014); Dad et al. (2015)	MLPA	19	No	Ger
40	*USH2A*	c.4314delG	p.Ile1439Tyrfs*15	‐‐‐	nov	SaS	c.4314delG	p.Ile1439Tyrfs*15	‐‐‐	x	nov	SaS	17	Yes	Asia
35	*USH2A*	c.4773delA	p.Glu1591Glufs*2	‐‐‐	nov	SaS	Del ex5‐11	Truncation	‐‐‐		nov	MLPA	30	No	Ger
112	*USH2A*	c.4933G>T	p.Gly1645*	‐‐‐	Sloan‐Heggen et al. (2016)	SaS	Del ex22‐24	Truncation	‐‐‐		Krawitz et al. (2014); Dad et al. (2015)	MLPA	33	No	Ger
84	*USH2A*	c.5776+1G>A	Splice	‐‐‐	Dreyer et al. (2008); Wang et al. (2014; Patel et al. (2016)	NGS	c.5776+1G>A	Splice	‐‐‐	x	Dreyer et al. (2008); Wang et al. (2014); Patel et al. (2016)	NGS	13	Yes	KSA
85	*USH2A*	c.7076T>G	p.Leu2359*	‐‐‐	Yang et al. (2013)	SaS	c.7595‐2144A>G	Splice	‐‐‐	x	Vache et al. (2012)	SaS	26	No	Ger
82	*USH2A*	c.7198delG	p.Asp2400Metfs*13	‐‐‐	nov	SaS	c.7198delG	p.Asp2400Metfs*13	‐‐‐	x	nov	SaS	42	No	Ger
129	*USH2A*	c.7595‐2144A>G	Splice	‐‐‐	Vache et al. (2012)	NGS	Del ex10‐11	Truncation	‐‐‐		Baux et al. (2014)	NGS	26	No	Ger
60	*USH2A*	c.8240delC	p.Pro2747Hisfs*22	‐‐‐	nov	SaS	c.13898delT	p.Leu4633*	‐‐‐		nov	SaS	71	No	Ger
126	*USH2A*	c.8834G>A	p.Trp2945*		McGee et al. (2010)	SaS	c.10561T>C	p.Trp3521Arg	rs111033264		Dreyer et al. (2008); McGee et al. (2010)	SaS	34	No	Ger
107*	*USH2A*	c.9258+1G>A	Splice	‐‐‐	nov	SaS	c.7595‐2144A>G	Splice	‐‐‐	x	Vache et al. (2012)	SaS	1	No	Ger
98	*USH2A*	c.8682‐9A>G	Splice	‐‐‐	Dreyer et al. (2008); Glockle et al. (2014)	SaS	c.12525G>A	p.Trp4175*	‐‐‐	x	Yan et al. (2009)	SaS	34	No	Ger
115	*USH2A*	c.8915delC	p.Ser2972Phefs*2	‐‐‐	nov	SaS	c.12234_12235delGA	p.Asn4079Trpfs*19	rs398124618		Baux et al. (2007)	SaS	25	No	Ger
38	*USH2A*	c.9424G>T	p.Gly3142*	‐‐‐	Baux et al. (2007)	SaS	c.9424G>T	p.Gly3142*	‐‐‐		Baux et al. (2007)	SaS	31	No	Ger
64	*USH2A*	c.9676C>T	p.Arg3226*	‐‐‐	Katagiri et al. (2014)	NGS	c.7595‐2144A>G	Splice	‐‐‐	x	Vache et al. (2012)	SaS	21	No	Ger
86	*USH2A*	c.9815C>T	p.Pro3272Leu	‐‐‐	Herrera et al. (2008)	SaS	c.5607_5615del	p.Arg1870_Ala1872del	‐‐‐		Krawitz et al. (2014)	NGS	44	No	Ger
114	*USH2A*	c.6928A>C	p.Thr2310Pro	‐‐‐	Le Quesne Stabej et al. (2012)	SaS	c.11864G>A	p.Trp3955*	rs111033364	x	van Wijk et al. (2004); Le Quesne Stabej et al. (2012)	NGS	16	No	Ger
62	*USH2A*	c.10388‐1G>A	Splice	‐‐‐	nov	SaS	c.11054G>A	p.Trp3685*	‐‐‐		nov	SaS	31	No	Ger
69	*USH2A*	c.10388‐1G>A	Splice	‐‐‐	nov	SaS	c.11054G>A	p.Trp3685*	‐‐‐		nov	SaS	19	No	Ger
66	*USH2A*	c.10759C>T	p.Gln3587*	rs111033418	Garcia‐Garcia et al. (2011)	NGS	c.11549‐1G>A	Splice	‐‐‐		Lenassi et al. (2015a,b)	NGS	12	No	Ger
111	*USH2A*	c.11065C>T	p.Arg3689*	rs41314534	Le Quesne Stabej et al. (2012); Aparisi et al. (2014)	SaS	c.12234_12235delGA	p.Asn4079Trpfs*19	rs398124618	x	Baux et al. (2007)	SaS	36	No	Ger
22	*USH2A*	c.11864G>A	p.Trp3955*	rs111033364	van Wijk et al. (2004); Le Quesne Stabej et al. (2012)	SaS	c.775_776delAG	p.Ser259Phefs*63	‐	x	Seyedahmadi et al. (2004)	SaS	68	No	Ger
17	*USH2A*	c.11864G>A	p.Trp3955*	rs111033364	van Wijk et al. (2004); Le Quesne Stabej et al. (2012)	SaS	c.1036A>C	p.Asn346His	rs369522997		Weston et al. (2000); Sadeghi et al. (2013); Wang et al. (2014); Lenassi et al. (2015a,b)	SaS	38	No	Ger
81	*USH2A*	c.11864G>A	p.Trp3955*	rs111033364	van Wijk et al. (2004); Le Quesne Stabej et al. (2012)	SaS	c.1271delT	p.Met424Argfs*34	‐‐‐		nov	SaS	14	No	Ger
106	*USH2A*	c.11864G>A	p.Trp3955*	rs111033364	van Wijk et al. (2004); Le Quesne Stabej et al. (2012)	SaS	c.1807G>A	p.Gly603Arg	‐‐‐	x	nov	SaS	30	No	Ger
24	*USH2A*	c.11864G>A	p.Trp3955*	rs111033364	van Wijk et al. (2004); Le Quesne Stabej et al. (2012)	SaS	c.2299delG	p.Glu767Serfs*21	rs80338903		Eudy et al. (1998)	SaS	45	No	Ger
25	*USH2A*	c.11864G>A	p.Trp3955*	rs111033364	van Wijk et al. (2004); Le Quesne Stabej et al. 2012)	SaS	c.2299delG	p.Glu767Serfs*21	rs80338903		Eudy et al. (1998)	SaS	17	No	Ger
83	*USH2A*	c.11864G>A	p.Trp3955*	rs111033364	van Wijk et al. (2004); Le Quesne Stabej et al. (2012)	SaS	c.4102C>T	p.Pro1368Ser	‐‐‐	x	nov	SaS	19	No	Ger
23	*USH2A*	c.11864G>A	p.Trp3955*	rs111033364	van Wijk et al. (2004); Le Quesne Stabej et al. (2012)	SaS	c.6642dupT	p.Leu2215Serfs*16	‐‐‐		nov	SaS	43	No	Ger
26	*USH2A*	c.11864G>A	p.Trp3955*	rs111033364	van Wijk et al. (2004); Le Quesne Stabej et al. (2012)	SaS	c.9270C>A	p.Cys3090*	‐‐‐		McGee et al. (2010)	SaS	21	No	Ger
48	*USH2A*	c.11864G>A	p.Trp3955*	rs111033364	van Wijk et al. (2004); Le Quesne Stabej et al. (2012)	SaS	c.11048‐2A>G	Splice	‐‐‐		nov	SaS	77	No	Ger
19	*USH2A*	c.11864G>A	p.Trp3955*	rs111033364	van Wijk et al. (2004); Le Quesne Stabej et al. (2012)	SaS	c.11549‐1G>A	Splice	‐‐‐		Lenassi et al. (2015a,b)	SaS	26	No	Ger
18	*USH2A*	c.11864G>A	p.Trp3955*	rs111033364	van Wijk et al. (2004); Le Quesne Stabej et al. (2012)	SaS	c.11864G>A	p.Trp3955*	rs111033364		van Wijk et al. (2004); Le Quesne Stabej et al. (2012)	SaS	41	n.d.	Turkey
49	*USH2A*	c.11864G>A	p.Trp3955*	rs111033364	van Wijk et al. (2004); Le Quesne Stabej et al. (2012)	NGS	c.11864G>A	p.Trp3955*	rs111033364		van Wijk et al. )^2004^); Le Quesne Stabej et al. (2012)	SaS	41	n.d.	Rus
20	*USH2A*	c.11864G>A	p.Trp3955*	rs111033364	van Wijk et al. (2004); Le Quesne Stabej et al. (2012)	SaS	Dup ex4	Truncation?	‐‐‐		nov	MLPA	49	No	Ger
16	*USH2A*	c.11864G>A	p.Trp3955*	rs111033364	van Wijk et al. (2004); Le Quesne Stabej et al. (2012)	SaS	Del ex22‐24	Truncation	‐‐‐		Krawitz et al. (2014); Dad et al. (2015)	MLPA	47	No	Ger
113	*USH2A*	c.11864G>A	p.Trp3955*	rs111033364	van Wijk et al. (2004); Le Quesne Stabej et al. (2012)	SaS	Del ex22‐24	Truncation	‐‐‐	x	Krawitz et al. (2014); Dad et al. 2015)	MLPA	34	No	Ger
80	*USH2A*	c.12067‐2A>G	Splice	‐‐‐	Auslender et al. (2008); Aparisi et al. (2014)	NGS	c.12067‐2A>G	Splice	‐‐‐		Auslender et al. (2008); Aparisi et al. (2014)	NGS	47	No	Jewish M‐Asia
105	*USH2A*	c.13010C>T	p.Thr4337Met	‐‐‐	Aller et al. (2006); Besnard et al. (2014); Ge et al. (2015)	SaS	c.14439_14454del	p.Cys4813*	‐‐‐		Koparir et al. (2015)	NGS	21	No	Ger
77	*USH2A*	c.14439_14454del	p.Cys4813*	‐‐‐	Koparir et al. (2015)	NGS	c.14439_14454del	p.Cys4813*	‐‐‐		Koparir et al. (2015)	NGS	44	n.d.	Turkey
91	*USH2A*	c.15017C>T	p.Thr5006Met	‐‐‐	Huang et al. (2015)	NGS	c.15017C>T	p.Thr5006Met	‐‐‐		Huang et al. (2015)	NGS	37	Yes	KSA
61	*USH2A*	Del ex14	Truncation	‐‐‐‐	Aparisi et al. (2014); Garcia‐Garcia et al. (2014); Glockle et al. (2014)	MLPA	Del ex14	Truncation	‐‐‐‐	x	Aparisi et al. (2014); Garcia‐Garcia et al. (2014); Glockle et al. (2014)	MLPA	21	n.d.	Syria
120	*USH2A*	Del ex45‐47	Truncation	‐‐‐	Baux et al. (2014)	NGS	Del ex45‐47	Truncation	‐‐‐		Baux et al. (2014)	NGS	25	Yes	Syria
39	*USH2A*	Del ex48	Truncation	‐‐‐	Neveling et al. (2013)	SaS, MLPA	Del ex48	Truncation	‐‐‐	x	Neveling et al. (2013)	SaS, MLPA	21	n.d.	Turkey
133	*ADGRV1*	c.7606G>T	p.Glu2536*	‐‐‐	nov	NGS	c.7606G>T	p.Glu2536*	‐‐‐		nov	NGS	28	n.d.	Ger
124	*ADGRV1*	c.8749G>T	p.Glu2917*	‐‐‐	nov	NGS	Del ex85	Truncation	‐‐‐		nov	NGS	45	No	Ger
45	*ADGRV1*	c.15716delA	p.Asn5239Thrfs*19	‐‐‐	nov	NGS	c.17204+5G>C	Splice	‐‐‐		nov	NGS	17	No	Ger
**USH2**	**Monoallelic**														
27	*USH2A*	c.2299delG	p.Glu767Serfs*21	rs80338903	Eudy et al. (1998)	SaS, NGS	‐‐‐	‐‐‐	‐‐‐		‐‐‐	SaS	50	No	Ger
119	*USH2A*	c.2299delG	p.Glu767Serfs*21	rs80338903	Eudy et al. (1998)	SaS, NGS	‐‐‐	‐‐‐	‐‐‐	x	‐‐‐	SaS, NGS	55	No	Ger
29	*USH2A*	c.2522C>A	p.Ser841Tyr	rs111033282	Jaijo et al. (2010); Garcia‐Garcia et al. (2011)	SaS, NGS	‐‐‐	‐‐‐	‐‐‐		‐‐‐	SaS, NGS	59	No	Ger
128	*USH2A*	c.8682‐9A>G	Splice	‐‐‐	Dreyer et al. (2008); Glockle et al. (2014)	SaS, NGS	‐‐‐	‐‐‐	‐‐‐		‐‐‐	SaS, NGS	35	No	Ger
65	*ADGRV1*	c.12895C>T	p.Arg4299*	‐‐‐	nov	NGS	‐‐‐	‐‐‐	‐‐‐		‐‐‐	NGS	27	No	Tunisia
92	*ADGRV1*	c.11410C>T	p.Arg3804*	‐‐‐	nov	NGS	‐‐‐	‐‐‐	‐‐‐		‐‐‐	NGS	25	n.d.	KSA
															
**USH2**	**Unsolved**														
55	‐‐‐	‐‐‐	‐‐‐	‐‐‐	‐‐‐	SaS/NGS	‐‐‐	‐‐‐	‐‐‐		‐‐‐	SaS/NGS	55	No	Ger
70	‐‐‐	‐‐‐	‐‐‐	‐‐‐	‐‐‐	SaS, NGS	‐‐‐	‐‐‐	‐‐‐		‐‐‐	SaS, NGS	50	No	Ger
74	‐‐‐	‐‐‐	‐‐‐	‐‐‐	‐‐‐	NGS	‐‐‐	‐‐‐	‐‐‐		‐‐‐	NGS	50	No	Ger

Pat, patient number; Ref, reference from the literature; Met, applied method(s); add. allele, heterozygous mutation in a secondary locus (in most cases reflecting carriership for a recessive mutation); nov, novel mutation (not previously reported); m, months; *, no retinal dystrophy diagnosed at the time the genetic diagnosis was made; SaS, Sanger sequencing; *PEX1*/*PEX6*, these genes were sequenced by the Sanger method before targeted panel‐NGS was applied; MS, locus‐specific polymorphic microsatellite markers for the known USH1 genes were genotyped. GLA, SNP‐array‐based genome‐wide linkage analysis. S, Segregation analysis performed. Ger, German; KSA, Kingdom of Saudi Arabia; Pak, Pakistan; Per, Persia; Sri L., Sri Lanka. In case of more than two potentially pathogenic alleles, the least likely causative one is in brackets.

### Workflow of genetic analysis and determination of diagnostic yield

The analytic workflow depended on the assumed diagnosis and the request of the physician in charge of the patient. If the clinical diagnosis was USH2, Sanger sequencing (and possibly MLPA) of the *USH2A* exons was the initial step of genetic testing in most cases because of the high probability to identify the causative mutation with this approach, followed by NGS for patients without *USH2A* mutations. For most patients who were categorized as USH1 or atypical Usher syndrome, NGS was carried out without other precedent tests. In P135, whose symptoms indicated a peroxisome biogenesis disorder (PBD), Sanger sequencing of *PEX1* and *PEX6* was carried out, followed by NGS. MLPA or array‐CGH analysis was conducted to verify CNVs that were indicated by quantitative analysis of NGS data (see below). In a few cases, genotyping of Usher locus‐specific polymorphic microsatellite markers or genome‐wide linkage analysis (as reported previously (Zaki et al. 2016)) preceded gene analysis.

When calculating the diagnostic yield, we considered patients with monoallelic mutations in a gene compatible with the respective clinical subtype as “resolved”, assuming that the secondary mutations had escaped detection due to atypical extra‐exonic localizations (deep‐intronic, non‐coding regulatory regions).

### Next‐generation sequencing (NGS)

Targeted next‐generation sequencing (NGS) was conducted for 112 genes (1914 coding exons) that have been associated with non‐syndromic (NSHL) and selected forms of syndromic hearing loss (SHL), including 11 genes associated with Usher syndrome (*MYO7A*/*USH1B*;* USH1C*;* CDH23*/*USH1D*;* PCDH15*/*USH1F*;* USH1G*;* CIB2*/*USH1J*;* USH2A*;* ADGRV1*/*USH2C*;* WHRN*/*USH2D*;* CLRN1*/*USH3A*;* PDZD7*/*USH2A* modifier, digenic contributor) and 14 linked to peroxisome biogenesis disorders (Table S1; including GenBank Accession Numbers of the wild‐type gene sequences), on a MiSeq or a HiSeq1500 system (Illumina), as previously described (Eisenberger et al. 2014). In brief, sheared DNA was ligated to barcoded adaptors for multiplexing. Exons were targeted by an in‐solution customized sequence capture library (NimbleGen). Amplified enriched DNA was subjected to NGS. Reads were mapped against the hg19 human reference genome using BWA (Li and Durbin 2009) and processed with SAMtools (Li et al. 2009), Picard (http://picard.sourceforge.net), and GATK (McKenna et al. 2010). Variants were filtered against dbNSFP v2.0 (Liu et al. 2011), dbSNP v137, the Human Gene Mutation Database (HGMD^®^ Professional 2013.2) (Stenson et al. 2014), and our in‐house database. The cutoff for the maximum minor allele frequency (MAF) was set to 1% (Bamshad et al. 2011). Nonsense, frameshift, and canonical splice site variants were regarded likely pathogenic. SNVs were assessed using SIFT (Ng and Henikoff 2003), MutationTaster (Schwarz et al. 2010), PolyPhen‐2 (Adzhubei et al. 2013), AlignGVGD (Mathe et al. 2006; Tavtigian et al. 2006), Pmut (Ferrer‐Costa et al. 2005), NNSPLICE v0.9 (Reese et al. 1997), and NetGene2 (Brunak et al. 1991; Hebsgaard et al. 1996). SeqPilot SeqNext module (v4.0.1, JSI medical systems) was used for visualization and final assessment of SNVs. Verification of all point mutations identified by NGS was carried out by Sanger sequencing. If samples from other family members were available, segregation analyses were carried out to confirm biallelic constellations – in particular in case of compound‐heterozygous mutations, but also in case of apparent homozygosity to rule out large deletions *in trans* to point mutations. Because the identified mutations were clearly pathogenic in almost all cases, biallelic situations are very likely true also in cases where segregation analyses were not possible.

### Copy number variation analysis from NGS data

We performed copy number variation (CNV) analysis on highly covered samples sequenced on the Illumina Hiseq1500^TM^ system. Potential copy number alterations (CNA) were initially identified with the tools copy number and copyCaller from VarScan v2.3.6 (Koboldt et al. 2012) on mapped reads with a maximum segment size of 300. All other parameters were used with standard settings. Thereby, coverage of every target region of the sample of interest was internally normalized and compared versus normalized control data of other samples of the same run. CNVs were annotated using RefSeq gene file from UCSC (ftp://hgdownload.cse.ucsc.edu/golden-Path/hg19/database/refGene.txt.gz). CNVs were initially taken into account if indicated by VarScan against at least 85% of the control patients and if the log_2_ threshold was ≥0.6 (in case of an amplification) or ≤−0.6 (in case of a deletion).

### MLPA and array‐CGH

Results from CNV analysis were verified by MLPA (multiplex ligation‐dependent probe amplification) analysis or, if corresponding MLPA kits were not available, by array‐CGH. The following SALSA MLPA probe mixes (MRC‐Holland, Amsterdam, The Netherlands) were applied: P361‐A1 and P362‐A1 for *USH2A*, and P292‐A2 for *PCDH15* (*USH1F*). In every MLPA analysis, six samples without CNVs in the investigated locus were used as negative controls.

Molecular karyotyping (array‐CGH) was performed using Agilent Human Genome CGH 244A (Agilent Technologies, Santa Clara, CA, USA) according to the manufacturer's instructions. Genomic positions were defined using NCBI37/hg19. CNVs were considered if at least five contiguous oligonucleotides presented with an abnormal log_2_ ratio.

### Translational read‐through approach for p.Trp3955*_USH2A_

HEK293T cells (cultured at 37°C and 5% CO_2_ in Dulbecco's Modified Eagle Medium with GlutaMax™, with 10% fetal bovine serum; Invitrogen, Karlsruhe, Germany) were transiently transfected (Lipofectamine™ with PLUS™ reagent; Invitrogen, Karlsruhe, Germany) with cDNAs coding for the FN3 domains 24 and 35, the transmembrane domain, and the cytoplasmic tail (residues p.3955‐4175 fused to residues p.4926‐5202) of wild‐type and mutant *USH2A* (USH2A_p.Trp3955*), respectively. A cDNA fragment from c.12250‐15996 of *USH2A* isoform b, encoding protein residues p.3955‐5202, was amplified and inserted into the pDest SP S/F‐C‐Tag vector with a C‐terminal Flag tag. The region of c.12910–14988 was deleted using the restriction enzymes BlpI and PmII (NEB, Frankfurt am Main, Germany). The reading frame was recovered by insertion of the bases G and C at position c.12907 of the wild‐type sequence using the QuickChange Lightning Site‐Directed Mutagenesis Kit (Stratagene, La Jolla, CA). The p.Trp3955* mutation was generated using the QuickChange Lightning Site‐Directed Mutagenesis Kit.

After 6 h, PTC124 (Selleckchem, Houston, USA; dissolved in DMSO; Sigma‐Aldrich, Deisenhofen, Germany) was applied to the culture media for 48 hours. Read‐through of the nonsense mutation was validated by indirect immunofluorescence using antibodies against Flag (Sigma‐Aldrich) on methanol‐fixed HEK293T cells as previously described (Goldmann et al. 2012). The amount of restored USH2A protein expression was calculated as the ratio of Flag‐positive cells in PTC124‐treated p.Trp3955*‐transfected cells, normalized to the total amount of analyzed cells.

Cell cultures were grown on sterile cover slips and fixed using cold methanol. PBS‐washed cover slips were blocked with blocking solution (0.5% cold water fish gelatin, 0.1% ovalbumin in PBS) for 30 min, followed by incubation with primary antibodies overnight at 4°C. Cover slips were incubated with secondary antibodies conjugated to Alexa 488 (Molecular Probes, Leiden, Netherlands) and DAPI (4′,6‐diamidino‐2‐phenylindole, Sigma–Aldrich) for staining of the nuclear DNA for 1 h at room temperature. PBS‐washed cover slips were mounted in Mowiol 4.88 (Hoechst, Frankfurt, Germany).

## Results

### High diagnostic yield with predominance of Usher syndrome mutations

Biallelic mutations (*in trans* constellation was either proven by segregation analysis or very likely, see Methods) were identified in the vast majority of patients (97% of USH1, 90% of USH2, and 92% of atypical Usher syndrome). When considering patients with monoallelic mutations (USH1: none; USH2: six; atypical Usher: one) as resolved, the diagnostic yield was 97% for both USH1 and USH2, and 92% for atypical Usher syndrome. In one USH1 patient and in three USH2 patients, no mutation was identified despite NGS of the aforementioned extended gene panel. The genetic diagnosis was made before onset of RP in 10 young patients with apparently isolated hearing impairment: nine with Usher syndrome due to mutations in *MYO7A* and *USH2A*, and one with a peroxisome biogenesis disorder (PBD) due to compound heterozygous *PEX26* mutations. Overall, 83 alleles carried a novel mutation, several of which were observed more than once. This was often the case in patients from the KSA and other MENA countries, then often in homozygous state. The “rarest” Usher syndrome genes with mutations were as follows: *USH1G* (1 patient), *CLRN1* (1 patient), *PCDH15* (2 patients), and *USH1C* (4x).

### CNVs account for a significant proportion of Usher syndrome mutations

**Figure 2:**
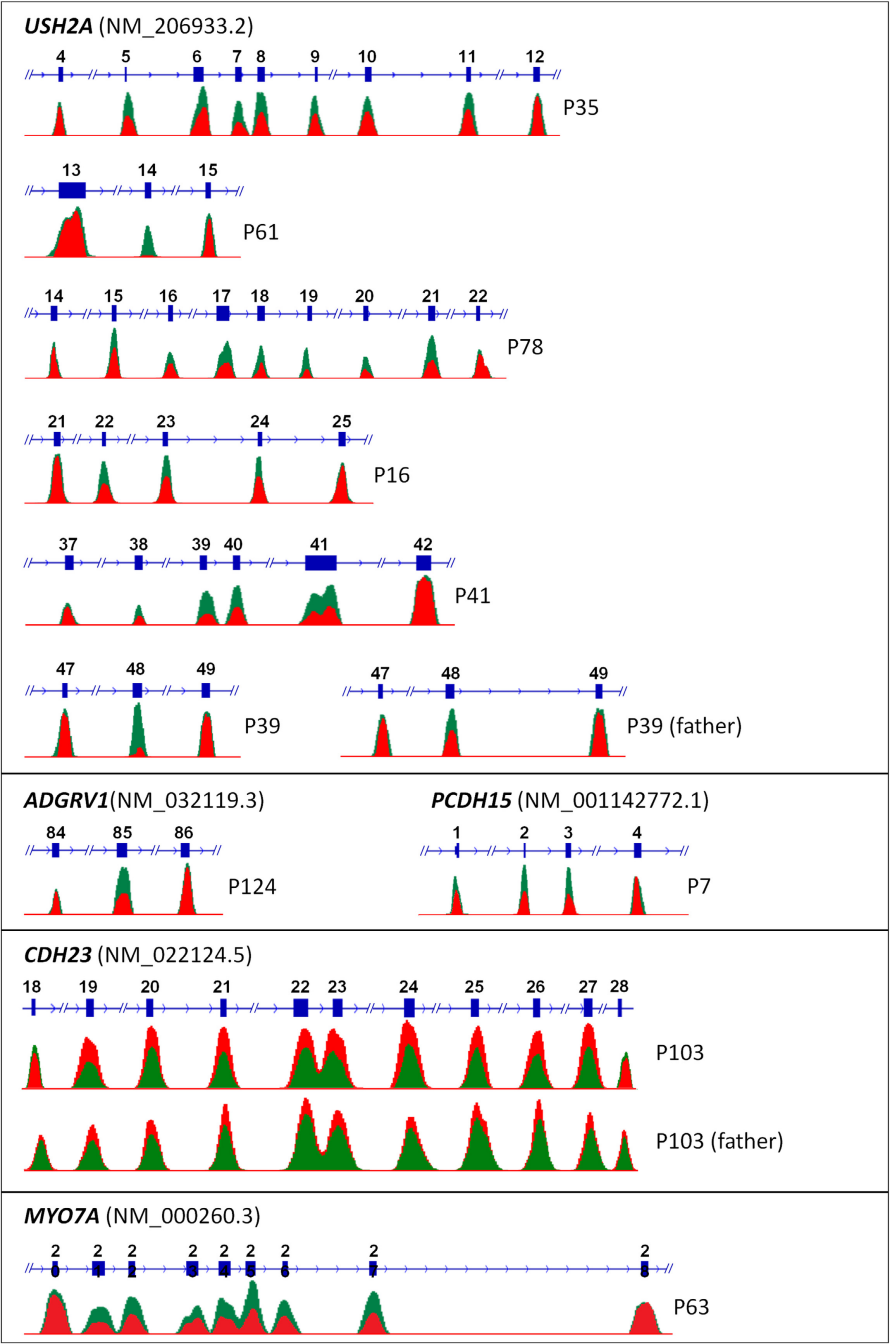
CNVs in USH genes detected by quantitative analysis of NGS data. The coverage plots illustrate the statistical readout, with the absolute coverage deduced from unique read count and as calculated by the CNV analysis mode in SeqNext (JSI Medical Systems). The coverage of affected and neighboring exons of patients (red) and controls (green) from the same NGS runs is shown in overlay schemes for comparison. While most patients harbor heterozygous deletions, reflected by approximately 50% reduction in coverage, patients P61 and P39 (the heterozygous father is shown for comparison) have homozygous deletions, reflected by virtually no coverage in the respective plot. Patient P103 had a homozygous duplication of nine *CDH23* exons (19‐27; also see Fig. S1) the heterozygous father is depicted for comparison.

We have established quantitative analysis of NGS data to detect CNVs such as deletions or duplications of one or several exons. We have previously shown that this bioinformatic tool effectively uncovers such structural mutations which escape detection in conventional approaches (PCR and Sanger sequencing of exons) if present in heterozygous state (Eisenberger et al. 2013). In our cohort, CNVs in USH genes significantly contribute to the mutational load. Compatible with its prevalence, but probably also due to its large size, *USH2A* is most often affected (Fig. 2). In patients with biallelic *USH2A* mutations, CNVs account for 10% (16/157 alleles; Table 1, Fig. 4A). Some CNVs were observed more than once and likely represent regional founder alleles. For example, a deletion of *PCDH15* exons 1–3 was found in two families from Syria.

**Figure 3:**
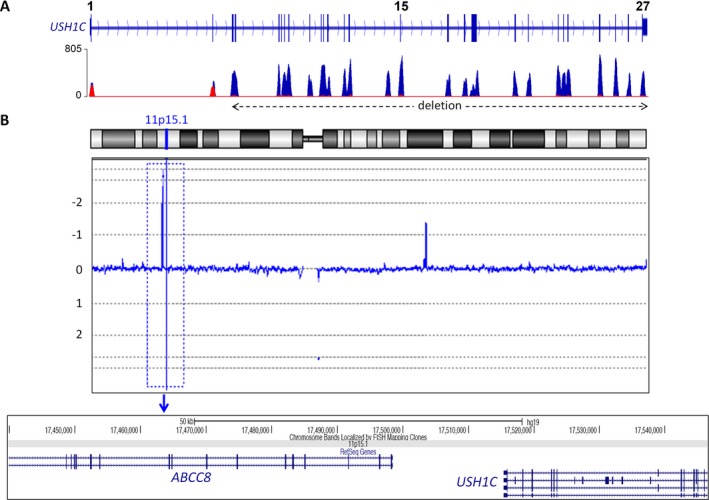
Contiguous gene syndrome due to a deletion of *USH1C* and *ABCC8*. (A) NGS indicated a homozygous deletion of *USH1C* exons 3–27 in two not knowingly related USH1 patients from Saudi Arabia, P96 and P97. (B) Array‐CGH revealed that the deletion also comprises the neighboring *ABCC8* gene. Thus, the alteration corresponds to a contiguous gene syndrome previously described in the *USH1C* gene identification study (Bitner‐Glindzicz et al. 2000). The replication of this mutation in our study indicates that this is a founder mutation from the Arabian Peninsula.

In two (not knowingly related) Saudi patients with Usher syndrome and hyperinsulinism (P96 and P97), we identified a homozygous deletion of the largest part of the *USH1C* gene (exons 3–27). One patient, P97, had a family history with likewise affected members and a deletion involving *USH1C* and *ABCC8*. Accordingly, high‐resolution array‐CGH revealed a homozygous microdeletion of approximately 123 kb on chromosome an 11p15.1 between genomic positions 17,439,772 and 17,546,526 bp (Fig. 3), defined by 14 contiguous oligomers (eight in *ABCC8*, MIM #600509; three in *USH1C*; three between *ABCC8* and *USH1C*). The deletion breakpoints are located in intron 22 of *ABCC8* and in intron 2 of *USH1C*, corresponding to the previously reported 11p15‐p14 deletion syndrome (MIM #606528 (Bitner‐Glindzicz et al. 2000)). In addition to Usher syndrome, patients with this condition present with congenital hyperinsulinism, severe enteropathy, and renal tubulopathy, and they may develop non‐autoimmune diabetes in adolescence (Hussain et al. 2004; Al Mutair et al. 2013). In P96, the *USH1C*/*ABCC8* deletion was primarily detected by NGS and confirmed by array‐CGH.

### p.Thr3977*_USH2A_: highly prevalent in USH2 and rectifiable by read‐through drugs

**Figure 4:**
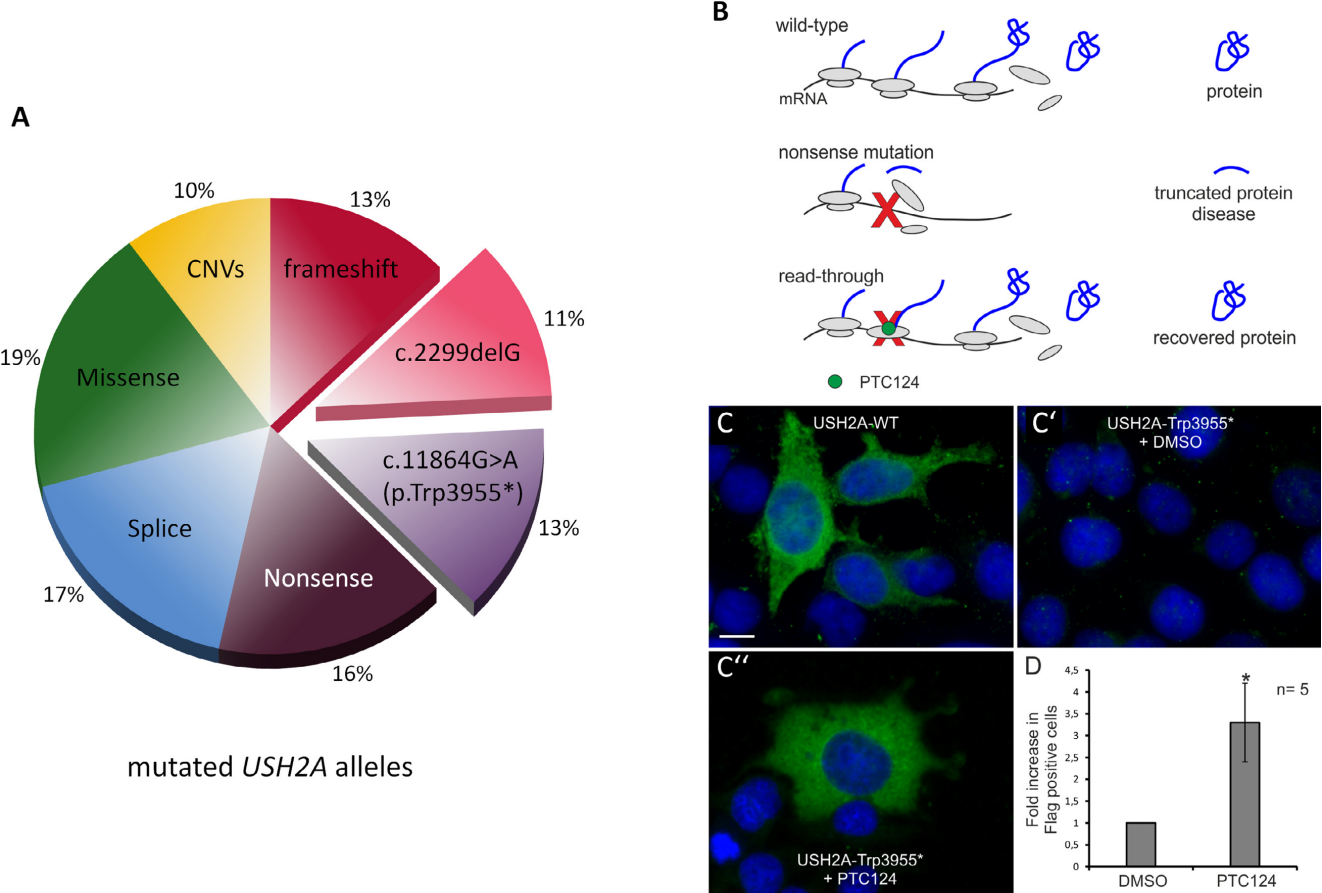
High prevalence of *USH2A* nonsense mutation p.Trp3955* and drug‐mediated read‐through. (A) *USH2A* alleles: Proportion of missense and small in‐frame alterations, truncating point mutations (nonsense, small deletions, and duplications), and large CNVs affecting one or more exons. Two mutations, p.Trp3955* and c.2299delG, are predominant. (B) Scheme of PTC124‐induced translational read‐through of a nonsense mutation. In the wild‐type situation, translation of mRNA results in functional full‐length protein. Nonsense mutations introduce a premature termination codon (red X) on the mRNA level, resulting in a truncated non‐functional protein. Read‐through‐inducing drugs like Ataluren (PTC124) bind to the ribosomes and promote the incorporation of an amino acid at the position of a PTC, resulting in the expression of full‐length protein. (C) Indirect immunofluorescence analyses of PTC124‐induced translational read‐through in cells transfected with wild‐type (WT) and mutant (Trp3955*) constructs (indirect immunofluorescence, anti‐Flag antibodies). Flag‐tagged USH2A (green) was detected in USH2A‐WT cells but not in (C′) DMSO‐treated USH2A‐p.Trp3955* cells. (C``) Application of PTC124 recovered USH2A expression in p.Trp3955*‐transfected cells. Nuclei were stained with DAPI (blue). (D) Increase in USH2A‐Flag‐positive cells after application of PTC124 (quantification of five independent experiments. Error bars represent SD; *<0.05; magnification bar: 10 *μ*m).

The *USH2A* mutation c.2299delG (p.Glu767Serfs*21) is the most prevalent USH mutation in several populations (Liu et al. 1999; Leroy et al. 2001; Pennings et al. 2004; Aller et al. 2006, 2010a,b; Dreyer et al. 2008). Unexpectedly, we found that the *USH2A* nonsense mutation, c.11864G>A (p.Trp3955*), previously reported in several studies (van Wijk et al. 2004; Le Quesne Stabej et al. 2012; Lenarduzzi et al. 2015), was even more common in our cohort, accounting for 13% of determined *USH2A* alleles (compared to 11% for c.2299delG; Fig. 4A). Both, c.2299delG and p.Trp3955*, have been annotated in dbSNP (rs80338903 and rs111033364, respectively), and c.2299delG has a higher minor allele frequency (MAF) than p.Trp3955* (0.07915 compared to 0.01071%; ExAC database), with no homozygotes annotated in the healthy population. Although our cohort consists of patients from diverse geographic regions and ethnic backgrounds, the largest group consists of patients of German descent (65%). The high prevalence of c.2299delG and p.Trp3955* is in accordance with the results of a recent large‐scale study on Usher syndrome (Bonnet et al. 2016). In contrast to that study, however, we found predominance of the p.Trp3955* mutation in German patients where it exceeds the prevalence of c.2299delG.

### Read‐through of p.Trp3955*_USH2A_

The most prevalent mutation in our study, p.Trp3955* mutation in *USH2A*, alters the TGG codon at position 11864 of the cDNA sequence into a premature UAG stop codon. Targeting of such nonsense mutations by small molecules such as PTC124 (Welch et al. 2007), known as translational read‐through‐inducing drugs (TRIDs), has become an important therapy approach (Fig. 4B; reviewed in Nagel‐Wolfrum et al. (2016)). To test this approach for the *USH2A* p.Trp3955* nonsense mutation, we transfected HEK293T cells with Flag‐tagged wild‐type (*USH2A*‐WT) and mutant *USH2A* plasmids (*USH2A*‐Trp3955*), and determined USH2A expression by indirect immunofluorescence using anti‐Flag antibodies. In contrast to *USH2A*‐WT cells (Fig. 4C), a low number of Flag‐positive cells was detected in DMSO‐treated *USH2A*‐Trp3955* cells (Fig. 4C′), most probably resulting from spontaneous read‐through of the p.Trp3955* mutation. Application of PTC124 to the Trp3955* cells resulted in a 3.3‐fold increase of USH2A expression (Fig. 4C′′) compared to DMSO‐treated Trp3955* cells.

### Simultaneous homozygosity of mutations in non‐syndromic genes and a novel Heimler syndrome gene

**Figure 5:**
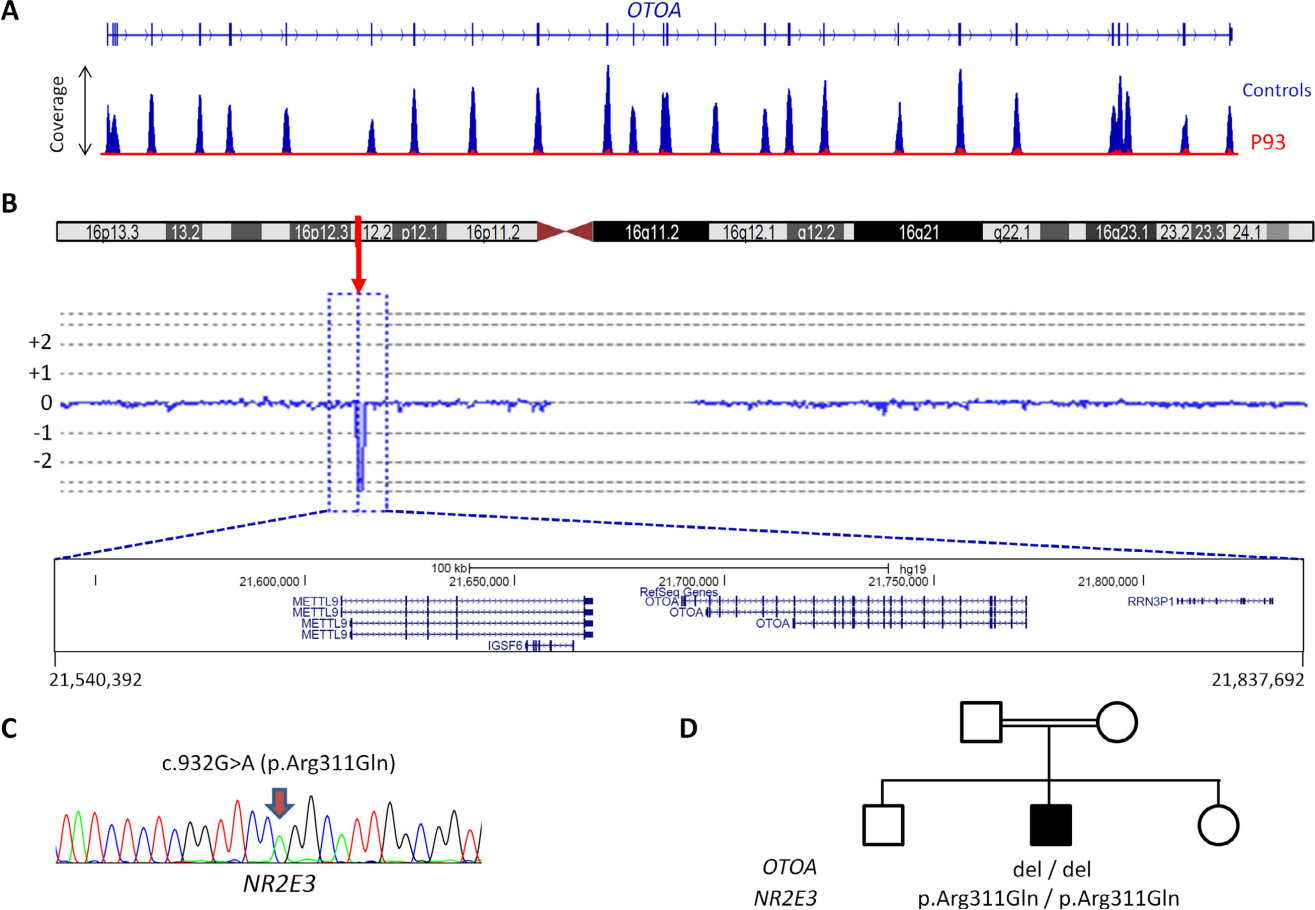
Double homozygosity for mutations in two genes associated with non‐syndromic disease simulates Usher syndrome in patient 93. (A) NGS indicated a homozygous deletion of the entire *OTOA* gene, the gene associated with recessive deafness *DFNB22*, in the patient. (B) This was confirmed by array‐CGH analysis. (C) Targeted analysis revealed a homozygous missense mutation of *NR2E3*. (D) Pedigree of the patient's consanguineous family summarizing the genetic findings.

In three patients with deaf‐blindness, disease was found to be due to “non‐Usher” gene mutations: Quantification of NGS reads in a Saudi patient from a consanguineous family, apparently affected by USH1, revealed a homozygous deletion of *OTOA*, a gene known to be associated with autosomal recessively inherited deafness, *DFNB22*. Because the patient's retinal phenotype (deep pigment deposits along the vascular arcades, subretinal fibrosis; delayed, depressed, and simplified scotopic flash response in the ERG) appeared compatible with a recessive *NR2E3*‐related dystrophy (Khan et al. 2007, 2010), this gene was sequenced. Indeed, a homozygous *NR2E3* missense mutation, p.Arg311Gln, previously reported as a pathogenic mutation (Haider et al. 2000; Kanda and Swaroop 2009; von Alpen et al. 2015), was identified (Fig. 5).

**Figure 6:**
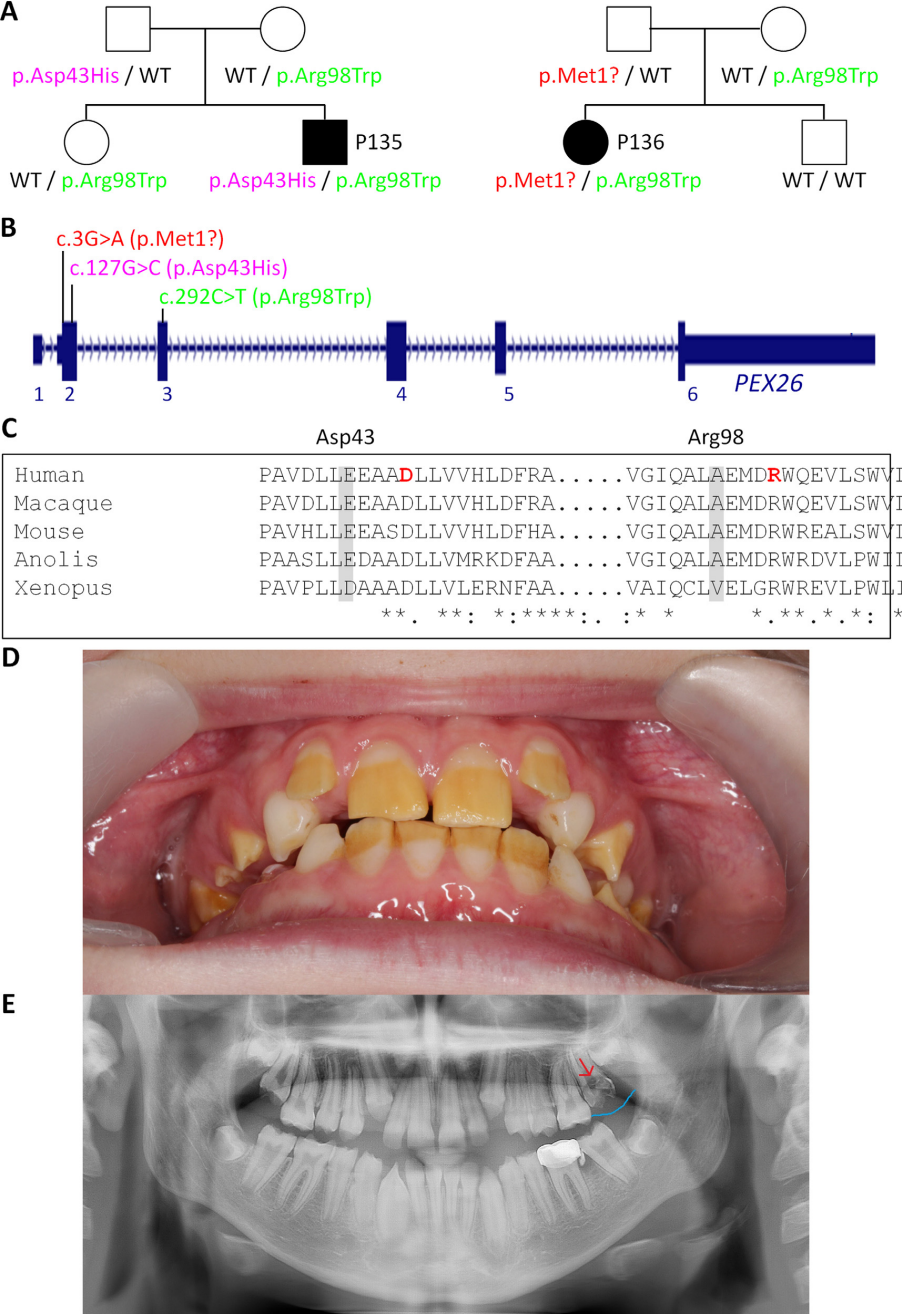
Biallelic *PEX26* mutations cause deaf‐blindness with enamel dysplasia (Heimler syndrome). (A) Pedigrees of the two patients with *PEX26* mutations. (B) Scheme of the *PEX26* gene and localization of mutations. (C) Partial alignment of PEX26/Pex26 peptide sequences from various species, indicating high evolutionary conservation of the mutated residues. (D) Severe enamel dysplasia of permanent teeth of patient 135 at 11^7^/_12_ years of age. (E) X‐ray of patient 135 showing preeruptive crown resorption in the upper left first molar (red arrow) and a local enlargement of the gingival tissue (blue line) at 13^10^/_12_ years of age.

Very recently, Heimler syndrome, characterized by the association of an “Usher‐like” presentation (retinal degeneration and hearing loss) with enamel dysplasia and nail abnormalities (Heimler et al. 1991), has been found to be caused by recessive mutations in two genes, *PEX1* and *PEX6*, known to be associated with peroxisome biogenesis disorders, PBD (Ratbi et al. 2015; Smith et al. 2016; Zaki et al. 2016). PBD‐associated genes have therefore been considered in our analysis. Here, we identified compound‐heterozygous mutations in another PBD‐related gene, *PEX26*, in two patients from two families (Fig. 6A): A 14‐year‐old boy (P135), diagnosed with “Usher syndrome with additional abnormalities”, carries two missense mutations affecting evolutionarily highly conserved residues, p.Asp43His and p.Arg98Trp (Fig. 6B,C). After birth, lack of reaction to noise was noted. At 22 months, profound hearing loss (80 dB), hepatosplenomegaly, and elevation of liver enzymes (which persisted) were diagnosed. Retinitis punctata albescens with macular involvement and significant visual loss was diagnosed at 5 ^1^/_2_ years. Opacities of deciduous teeth indicated thin enamel, and permanent teeth showed severe enamel dysplasia in terms of amelogenesis imperfecta combined with gingival hyperplasia and progressive preeruptive crown resorption (Fig. 6D,E). The other patient (P136), a 4‐year‐old girl with apparently non‐syndromic hearing loss, was found to carry a translation initiation codon mutation (p.Met1?) and, as P135, p.Arg98Trp. Subsequent detailed inspection of the deciduous teeth revealed enamel defects. Although development of retinal degeneration in this patient seems very likely, stressful in‐depth ophthalmological investigations were not carried out now.

## Discussion

Deaf‐blindness is mostly of genetic origin in developed countries, and biallelic mutations in the genes for Usher syndrome, an autosomal recessive disorder, are the predominant cause. With 11 known, mostly very large genes whose mutations explain the majority of cases, Usher syndrome is a prime condition for targeted NGS. Several reports have accordingly shown that the method has matured into a powerful diagnostic tool (Aparisi et al. 2014; Besnard et al. 2014; Bujakowska et al. 2014; Krawitz et al. 2014; Jiang et al. 2015; Bonnet et al. 2016).

The diagnostic yield in our study is very high and similar to a recent large‐scale study on Usher syndrome patients (Bonnet et al. 2016). Many mutations were novel, confirming that the genetic basis is often “private” and not detectable by screens that focus on previously reported mutations (Cremers et al. 2007). Repeatedly observed mutations, however, were common, not confined to but particularly in patients from MENA countries where the rate of parental consanguinity is high (Table 2). This indicates regional founder mutations that may guide the genetic diagnostic approach. Among the recurrent mutations are several large CNVs, including the 11p15‐p14 deletion syndrome that involves *USH1C* and *ABCC8* in two KSA families. Hyperinsulinism is therefore a symptom to be considered in deaf(‐blind) patients from the Arabian Peninsula.

**Table 2:** Repeatedly observed mutations

			Patients (n)	Origin
***MYO7A***				
c.397dupC	p.His133Profs*7		2	Pakistan
c.470+1G>A	Splice		2	KSA
c.2904G>T	p.Glu968Asp	rs111033233	2	Germany, Persia, Syria
c.3719G>A	p.Arg1240Gln	rs111033178	2	Germany
c.3503G>A	p.Arg1168Gln		2	Germany
***CDH23***				
c.6047‐9G>A	p.Leu728Serfs*6		2	Italy, Pakistan
***PCDH15***				
Del ex1‐3	p.Glu968Asp		2	Syria
***USH1C***				
Del ex3‐27	CNV		2	KSA
***USH2A***				
c.11864G>A	p.Trp3955*	rs111033364	19	Germany
c.2299delG	p.Glu767Serfs*21	rs80338903	16	Germany
Del ex22‐24	CNV		5	Germany
c.7595‐2144A>G	Splice		4	Germany
c.653T>A	p.Val218Glu		3	Germany
c.920_923dup	p.His308Glnfs*16		3	Germany
c.949C>A	p.Arg317Arg		3	Germany
c.1036A>C	p.Asn346His	rs369522997	3	Germany
c.8682‐9A>G	Splice		3	Germany
c.486‐1G>C	Splice		2	KSA
c.2209C>T	p.Arg737*	rs111033334	2	Germany
c.9424G>T	p.Gly3142*		2	Germany
c.10388‐1G>A	Splice		2	Germany
c.14439_14454del	p.Cys4813*		2	Germany, Turkey
c.14131C>T	p.Gln4711*		2	Germany
c.10561T>C	p.Trp3521Arg	rs111033264	2	Germany
c.12234_12235delGA	p.Asn4079Trpfs*19	rs398124618	2	Germany
c.11054G>A	p.Trp3685*		2	Germany
c.11549‐1G>A	Splice		2	Germany
***PEX26***				
c.292C>T	p.Arg98Trp	rs62641228	2	Germany

Here, we aimed at a one‐method approach, based on targeted NGS. To achieve this, we a) established quantitative readout of NGS data to detect CNVs, with conventional methods like MLPA and array‐CGH only being used for verification of NGS‐based CNV detection; b) included genes mutated in clinically overlapping conditions like Heimler syndrome; and c) simultaneously enriched genes implicated in isolated deafness to recognize cases with co‐occuring non‐syndromic defects mimicking a single syndrome.

Because all CNVs detected by quantitative readout of NGS data could be confirmed by MLPA or array‐CGH and the majority of patients were found to carry two (either proven or very likely) biallelic mutations, we assume that probably no CNV escaped detection in our study. This eliminates a major dead corner in diagnostic testing of deaf‐blindness genes and enables highly efficient testing by a *single* technique, targeted NGS comprising all exons of genes for Usher syndrome, clinically overlapping conditions, and the repeatedly reported deep‐intronic c.7595‐2144A>G mutation in *USH2A*. Deep‐intronic mutations apart from c.7595‐2144A>G_*USH2A*_ (which accounted for only four alleles in our study) have been reported (Liquori et al. 2016) and would still escape detection in our non‐genomic approach but – given the small proportion of patients with monoallelic or no mutations – do not seem to play a significant role. Very recently, however, by whole‐genome sequencing, we found evidence that a deep intronic founder mutation in *CLRN1* may significantly contribute to USH1 on the Arabian Peninsula (Khan et al. 2017). Such recurrent “hidden” splice mutations should be considered at least in patients with the respective ethnic background.

The clinical presentation of most patients with Usher syndrome corresponded to either USH1 or USH2. All genetically resolved patients from these two categories had mutations in Usher syndrome genes. The same applied to the nine patients diagnosed with “atypical Usher syndrome” in whom course and/or age of onset of sensorineural hearing impairment and RP did not allow for clear‐cut assignment to USH1 or USH2 and who had no additional abnormalities: They were found to have atypical expressions of USH1B (*MYO7A*), USH1D (*CDH23*), USH2A, and USH2C (*ADGRV1*). The diagnosis had to be reversed in the three remaining patients with apparent “atypical Usher syndrome”: They were found to have clinically similar conditions, a peroxisome biogenesis disorder (PBD; *PEX26* mutations), or simultaneous presence of two non‐syndromic conditions. Retrospectively, these patients had additional abnormalities (in case of *PEX26*‐associated PBD) or a distinct, *NR2E3*‐typical form of retinopathy. Detailed clinical characterization before genetic testing could have largely ruled out Usher syndrome beforehand. However, patients undergoing genetic testing for deaf‐blindness usually represent a heterogeneous cohort and range from poor to excellent clinically characterized. Although most will have mutations in Usher syndrome genes, it is the geneticists’ role to anticipate this problem and to equally take rare differential diagnoses into account.

In patient 93, the specific retinopathy due to *NR2E3* mutation homozygosity could have indicated a diagnosis distinct from Usher syndrome, but congenital deafness resulting from the homozygous deletion of *OTOA* is indistinguishable from the hearing impairment in USH1. If both components, deafness and RP, present as in Usher syndrome, as we have previously reported for patients with double homozygosity for mutations in *DFNB59* (deafness) and *MERTK* (RP) (Ebermann et al. 2007), and if the co‐occurence of the two non‐syndromic conditions in the index patient is not uncovered by their division in siblings, the genetic diagnosis is essential. Although the disentanglement of the genetic basis of disease in patient 93 does not change his medical follow‐up, it makes a major difference in genetic counseling for the parents whose family planning was ongoing: Instead of a 25% recurrence risk for Usher syndrome in further children, the actual risk is 25% for non‐syndromic deafness, 25% for non‐syndromic retinopathy, and 6.25% for deaf‐blindness. With the increasing diagnostic application of large‐scale panel NGS, whole‐exome and whole‐genome sequencing, it has become apparent that the co‐occurence of two (or more) monogenic conditions in one patient is not so uncommon (Boycott and Innes 2017) – and hard to recognize if it resembles a syndrome. Given the relatively high prevalence of carriers for (mostly recessively inherited) monogenic retinal dystrophies (with about 20% of the general population assumed to be carriers (Nishiguchi and Rivolta 2012)) and hearing impairment (assuming a monogenic cause in about 80% of the affected newborns (1 in 500) (Shearer and Smith 2012)), mimicking of Usher syndrome by both conditions should be a recurrent scenario. Although this is more likely in offspring from consanguineous parents (as in case of patient 93), migration from regions with high rates of consanguinity, like the Middle East and North Africa (MENA regions), this phenomenon will increase, for example, in Central Europe. In summary, our results demonstrate the potential of extended NGS panels that include non‐Usher genes to resolve difficult genetic constellations.

Inherited retinal dystrophies are a major cause of blindness worldwide. There has been remarkable progress in different therapeutic approaches (Scholl et al. 2016), such as gene therapy and optogenetics. Gene addition with adeno‐associated viral (AAV) vectors has shown to be effective in case of *RPE65* in patients with Leber's congenital amaurosis (LCA) type 2. In Usher syndrome, the retinopathy component would be the primary target of gene therapy, especially in patients who still have early‐stage RP or who are still non‐syndromic (“only” deaf). The identification of the causative gene will therefore be of utmost importance if gene therapy should become routinely available. However, the enormous size of many genes, including *USH2A*, represents a major hurdle for the packaging capacity of AAV vectors. Therefore, alternative gene‐based strategies for therapy or slowdown of retinal degeneration are necessary. Translational read‐through represents a promising alternative for patients with nonsense mutations (Nagel‐Wolfrum et al. 2014a,b). Here, we show that PTC124 effectively induces translational read‐through of the predominant p.Trp3955* *USH2A* nonsense mutation which accounted for 13% of determined mutant *USH2A* alleles in our cohort. Because 35% of Usher syndrome patients with determined mutations in our study carry nonsense mutations on at least one allele (48/138), translational read‐though could be a promising therapeutic strategy for Usher syndrome patients of all genetic subtypes.

Pinpointing the molecular diagnosis can be crucial for specific prophylaxis – as in case of patients P135 and P136 who both carry biallelic *PEX26* mutations. While P135 expresses the full picture of Heimler syndrome (except specific nail abnormalities which do not seem to be obligate part of the syndrome (Ratbi et al. 2016; Witters et al. 2016)), the only “Heimler sign” so far in patient P136 was enamel dysplasia, probably because of her young age (3 years). This patient can benefit, in terms of early prophylaxis, from the molecular diagnosis by being monitored for signs of hepatopathy, elevation of fatty acids and retinopathy. To protect hepatic function and lipid metabolism, the patient should avoid certain medications and noxious substances (e.g., alcohol). Furthermore, and in contrast to Usher syndrome, therapeutic options could exist for patients with mild PBD who may benefit from a phytanic acid‐restricted diet and extracorporeal lipid apheresis (Baldwin et al. 2010; Ruether et al. 2010; Kohlschütter et al. 2012). In the ideal case, an effective diet could also counteract progression or even manifestation of retinal degeneration. Nine patients (in whom biallelic Usher or, in one case, Heimler syndrome gene mutations were found) had been diagnosed with non‐syndromic deafness. If AAV‐based gene addition or read‐through approaches should become available as regular therapies, such early diagnosed patients could benefit immensely from their early genetic diagnosis.

While exome sequencing has become a reasonable “one‐test‐solution” for genetically heterogeneous conditions with a significant proportion of patients lacking mutations in the known disease genes, we propose NGS (large) panel analysis for Usher(‐like) syndrome – with the advantage of technically feasible CNV discovery, a very high diagnostic yield, and uncovering conditions mimicking Usher syndrome.

## Conflict of Interest

CN, TE, CD, SN, CBl, and HJB are employees of Bioscientia which is part of a publicly traded diagnostic company. The other authors have no competing interests.

## Supporting information


**Figure S1.** Confirmation of a heterozygous intragenic deletion of *CDH23* exons 19–27 in patient P103 by array‐CGH (244k Agilent Technologies microarray).

**Table S1.** Deafness genes analyzed by targeted NGS.
