# Supplementary figures and images for: Next‐generation sequencing reveals the mutational landscape of clinically diagnosed Usher syndrome: copy number variations, phenocopies, a predominant target for translational read‐through, and PEX26 mutated in Heimler syndrome
Source: Mol Genet Genomic Med. 2017 Jul 6;5(5):531–52. doi: 10.1002/mgg3.312 (PMC5606877; doi:10.1002/mgg3.312)

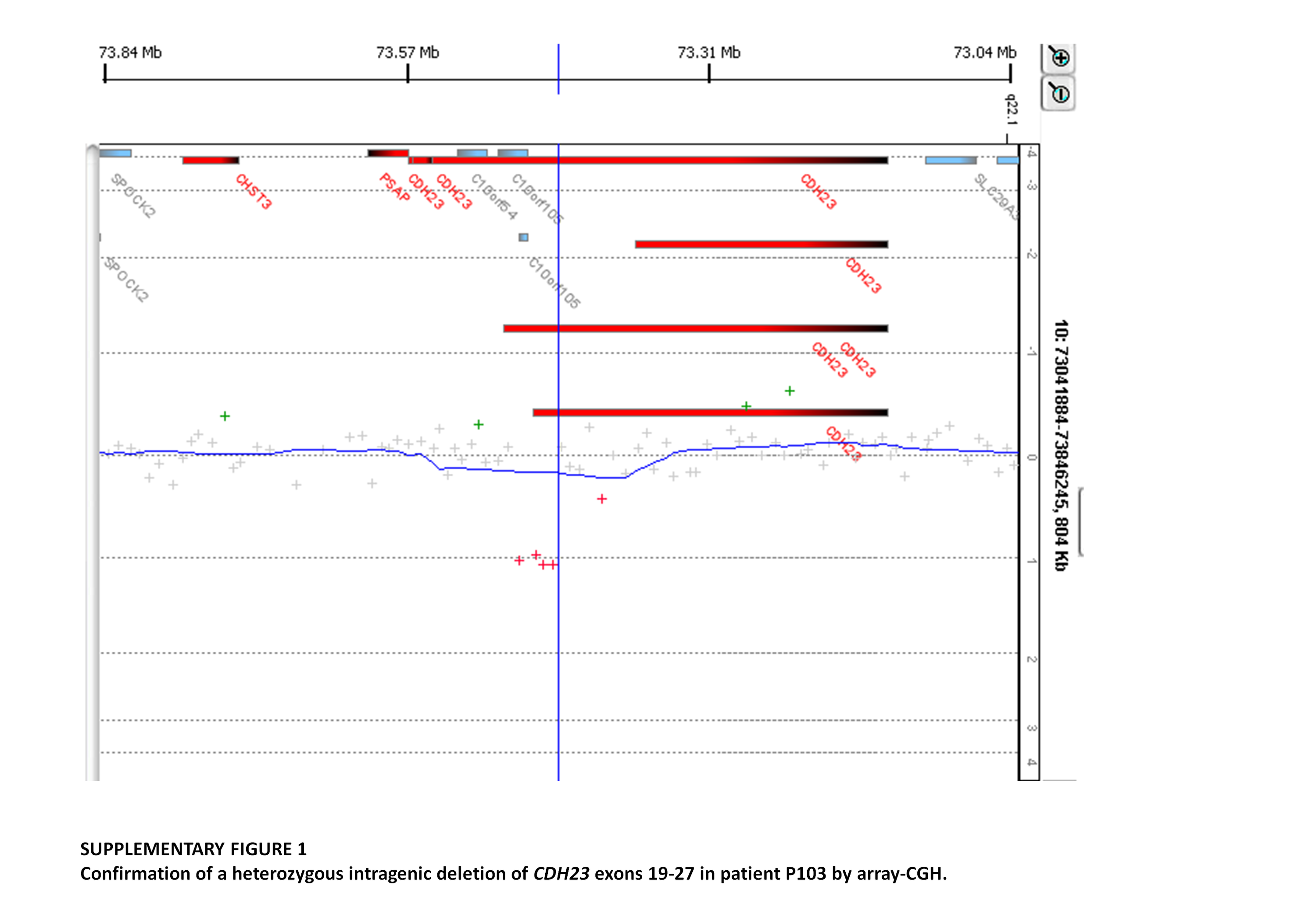

Supplement: Supplementary file 1 — Figure S1. Confirmation of a heterozygous intragenic deletion of CDH23 exons 19–27 in patient P103 by array‐CGH (244k Agilent Technologies microarray). [file MGG3-5-531-s001.TIF]
