# Supplementary material for: Next‐generation sequencing reveals the mutational landscape of clinically diagnosed Usher syndrome: copy number variations, phenocopies, a predominant target for translational read‐through, and PEX26 mutated in Heimler syndrome
Source: Mol Genet Genomic Med. 2017 Jul 6;5(5):531–52. doi: 10.1002/mgg3.312 (PMC5606877; doi:10.1002/mgg3.312)
Supplement: Supplementary file 2 — Table S1. Deafness genes analyzed by targeted NGS. [file MGG3-5-531-s002.docx]

**TABLE S1**

**Deafness genes analyzed by NGS in this study.** ARNSHL, autosomal recessive nonsyndromic hearing loss; ADNSHL, autosomal dominant nonsyndromic hearing loss; XLNHSL, X-linked nonsyndromic hearing loss; SHL, syndromic hearing loss. DFNA-, DFNB- and DFNX- designate loci for ADNSHL, ARNSHL and XLNSHL, respectively. "x", no locus designation. SANDD, sinoatrial node dysfunction and deafness; USH, Usher syndrome; PBD, peroxisome biogenesis disorder; PDS, Pendred syndrome; JLNS, Jervell and Lange-Nielsen syndrome; SeSAME, seizures, sensorineural deafness, ataxia, mental retardation and electrolyte imbalance; DDS, deafness dystonia syndrome. *STRC* could not be captured due to several homologous sequences in the genome.

| **Gene** | **ARNSHL** | **ADNSHL** | **XLNSHL** | **SHL** | **Modifier of** | **Main isoforms** | **Coding exons** |
| --- | --- | --- | --- | --- | --- | --- | --- |
| *ACTG1* |  | *DFNA20/26* |  |  |  | NM_001614.3 | 5 |
| *ADCY1* | *DFNB44* |  |  |  |  | NM_021116.2 | 20 |
| *ATP2B2* |  |  |  |  | *DFNB12, DFNA22* | NM_001001331.2 | 22 |
| *BDP1* | (x) |  |  |  |  | NM_018429.2 | 39 |
| *BSND* | *DFNB73* |  |  |  |  | NM_057176.2 | 4 |
| *CABP2* | *DFNB93* |  |  |  |  | NM_016366.2 | 7 |
| *CACNA1D* |  |  |  | *SANDD* |  | NM_000720.3; NM_001128840.2 | 50 |
| *CEACAM16* |  | *DFNA4B* |  |  |  | NM_001039213.3 | 6 |
| *CCDC50* |  | *DFNA44* |  |  |  | NM_178335.2 | 12 |
| *CDH23* | *DFNB12* |  |  | *USH1D* |  | NM_022124.5 | 67 |
| *CIB2* | *DFNB48* |  |  | *USH1J* |  | NM_006383.3 | 6 |
| *CLDN14* | *DFNB29* |  |  |  |  | NM_144492.2 | 1 |
| *CLIC5* | *DFNB103* |  |  |  |  | NM_001114086.1 | 6 |
| *CLRN1* |  |  |  | *USH3A* |  | NM_174878.2 | 3 |
| *COCH* |  | *DFNA9* |  |  |  | NM_001135058.1 | 11 |
| *COL11A2* |  | *DFNA13* |  |  |  | NM_080680.2 | 66 |
| *CRYM* |  | *x* |  |  |  | NM_001888.3 | 8 |
| *DCDC2* | *DFNB66* |  |  |  |  | NM_016356.4 | 10 |
| *DFNA5* |  | *DFNA5* |  |  |  | NM_004403.2 | 9 |
| *DFNB31* | *DFNB31* |  |  | *USH2D* |  | NM_015404.3 | 12 |
| *DFNB59* | *DFNB59* |  |  |  |  | NM_001042702.3 | 6 |
| *DIABLO* |  | *DFNA64* |  |  |  | NM_019887.5 | 6 |
| *DIAPH1* |  | *DFNA1* |  |  |  | NM_005219.4 | 28 |
| *DIAPH3* |  | *AUNA1* |  |  |  | NM_001042517.1 | 28 |
| *ELMOD3* | *DFNB88* |  |  |  |  | NM_032213.4 | 10 |
| *EPS8* | *DFNB102* |  |  |  |  | NM_004447.5 | 20 |
| *ESPN* | *DFNB36* | *x* |  |  |  | NM_031475.2 | 13 |
| *ESRRB* | *DFNB35* |  |  |  |  | NM_004452.3 | 8 |
| *EYA4* |  | *DFNA10* |  |  |  | NM_004100.4 | 19 |
| *FAM65B* | *DFNB104* |  |  |  |  | NM_014722.3 | 22 |
| *FOXI1* |  |  |  | *PDS* |  | NM_012188.4 | 2 |
| *GIPC3* | *DFNB15/72/95* |  |  |  |  | NM_133261.2 | 6 |
| *GJB2* | *DFNB1A* | *DFNA3A* |  |  |  | NM_004004.5 | 1 |
| *GJB3* | *x* | *DFNA2B* |  |  |  | NM_024009.2 | 1 |
| *GJB6* | *DFNB1B* | *DFNA3B* |  |  |  | NM_001110219.2 | 1 |
| *GPR98* |  |  |  | *USH2C* |  | NM_032119.3 | 90 |
| *GPSM2* | *DFNB82* |  |  |  |  | NM_013296.4 | 14 |
| *GRHL2* |  | *DFNA28* |  |  |  | NM_024915.3 | 16 |
| *GRXCR1* | *DFNB25* |  |  |  |  | NM_001080476.2 | 4 |
| *GRXCR2* | *DFNB101* |  |  |  |  | NM_001080516.1 | 3 |
| *HGF* | *DFNB39* |  |  |  |  | NM_000601.4 | 18 |
| *HOMER2* |  | x |  |  |  | NM_199330.2 | 9 |
| *ILDR1* | *DFNB42* |  |  |  |  | NM_001199799.1 | 8 |
| *KARS* | *DFNB89* |  |  |  |  | NM_001130089.1 | 14 |
| *KCNE1* |  |  |  | *JLNS2* |  | NM_001127668.2 | 1 |
| *KCNJ10* |  |  |  | *SeSAMES* |  | NM_002241.4 | 1 |
| *KCNQ1* |  |  |  | *JLNS1* |  | NM_000218.2 | 16 |
| *KCNQ4* |  | *DFNA2A* |  |  |  | NM_004700.3 | 14 |
| *KITLG* |  | *DFNA69* |  |  |  | NM_000899.4 | 9 |
| *LHFPL5* | *DFNB66/67* |  |  |  |  | NM_182548.3 | 3 |
| *LOXHD1* | *DFNB77* |  |  |  |  | NM_144612.6 | 40 |
| *LRTOMT* | *DFNB63* |  |  |  |  | NM_145309.5 | 4 |
| *MARVELD2* | *DFNB49* |  |  |  |  | NM_001038603.2 | 6 |
| *MIR96* |  | *DFNA50* |  |  |  | NR_029512.1 | 1 (fragment) |
| *MSRB3* | *DFNB74* |  |  |  |  | NM_198080.3 | 6 |
| *MYH14* |  | *DFNA4* |  |  |  | NM_001145809.1 | 42 |
| *MYH9* |  | *DFNA17* |  |  |  | NM_002473.4 | 40 |
| *MYO15A* | *DFNB3* |  |  |  |  | NM_016239.3 | 64 |
| *MYO3A* | *DFNB30* |  |  |  |  | NM_017433.4 | 33 |
| *MYO6* | *DFNB37* | *DFNA22* |  |  |  | NM_004999.3 | 34 |
| *MYO7A* | *DFNB2* | *DFNA11* |  | *USH1B* |  | NM_000260.3 | 48 |
| *OSBPL2* | *DFNA67* |  |  |  |  | NM_144498.2 | 13 |
| *OTOA* | *DFNB22* |  |  |  |  | NM_144672.3 | 28 |
| *OTOF* | *DFNB9* |  |  |  |  | NM_194248.2 | 46 |
| *OTOG* | *DFNB18B* |  |  |  |  | NM_001277269.1 | 55 |
| *OTOGL* | *DFNB84* |  |  |  |  | NM_173591.3 | 58 |
| *P2RX2* | *DFNA41* |  |  |  |  | NM_174873.2 | 12 |
| *PCDH15* | *DFNB23* |  |  | *USH1F* |  | NM_033056.3; NM_001142763.1; NM_001142771.1 | 35 |
| *PDZD7* |  |  |  | *USH2 (digenic)* | *USH2C* | NM_001195263.1; NM_024895.4 | 17 |
| *PEX1* |  |  |  | PBD |  | NM_000466.2 | 24 |
| *PEX2* |  |  |  | PBD |  | NM_000318.2 | 1 |
| *PEX3* |  |  |  | PBD |  | NM_003630.2 | 12 |
| *PEX5* |  |  |  | PBD |  | NM_001131025.1 | 15 |
| *PEX6* |  |  |  | PBD |  | NM_000287.3 | 17 |
| *PEX7* |  |  |  | PBD |  | NM_000288.3 | 10 |
| *PEX10* |  |  |  | PBD |  | NM_153818.1 | 6 |
| *PEX12* |  |  |  | PBD |  | NM_000286.2 | 3 |
| *PEX13* |  |  |  | PBD |  | NM_002618.3 | 4 |
| *PEX14* |  |  |  | PBD |  | NM_004565.2 | 9 |
| *PEX16* |  |  |  | PBD |  | NM_004813.2 | 11 |
| *PEX19* |  |  |  | PBD |  | NM_002857.3 | 8 |
| *PEX26* |  |  |  | PBD |  | NM_017929.5 | 5 |
| *PHYH* |  |  |  | PBD |  | NM_006214.3 | 9 |
| *PNPT1* | *DFNB70* |  |  |  |  | NM_033109.4 | 28 |
| *POU3F4* |  |  | *DFNX2* |  |  | NM_000307.4 | 1 |
| *POU4F3* |  | *DFNA15* |  |  |  | NM_002700.2 | 2 |
| *PRPS1* |  |  | *DFNX1* |  |  | NM_002764.3 | 7 |
| *PTPRQ* | *DFNB84* |  |  |  |  | NM_001145026.1 | 42 |
| *RDX* | *DFNB24* |  |  |  |  | NM_002906.3 | 13 |
| *SERPINB6* | *DFNB91* |  |  |  |  | NM_004568.5 | 6 |
| *SIX1* |  | *DFNA23* |  |  |  | NM_005982.3 | 2 |
| *SLC17A8* |  | *DFNA25* |  |  |  | NM_139319.2 | 12 |
| *SLC26A4* | *DFNB4* |  |  | *PDS* |  | NM_000441.1 | 20 |
| *SLC26A5* | *DFNB61* |  |  |  |  | NM_198999.2 | 18 |
| *SYNE4* | *DFNB76* |  |  |  |  | NM_001039876.2 | 8 |
| *TBC1D24* | *DFNB86* | *DFNA65* |  |  |  | NM_001199107.1 | 7 |
| *TECTA* | *DFNB21* | *DFNA8/12* |  |  |  | NM_005422.2 | 23 |
| *TIMM8A* |  |  | *DDS* |  |  | NM_004085.3 | 2 |
| *TJP2* |  | *DFNA51* |  |  |  | NM_004817.3 | 23 |
| *TMC1* | *DFNB7/11* | *DFNA36* |  |  |  | NM_138691.2 | 20 |
| *TMEM132E* | *DFNB99* |  |  |  |  | NM_001304438.1 | 9 |
| *TMIE* | *DFNB6* |  |  |  |  | NM_147196.2 | 4 |
| *TMPRSS3* | *DFNB8/10* |  |  |  |  | NM_024022.2 | 12 |
| *TNC* |  | *DFNA56* |  |  |  | NM_002160.3 | 27 |
| *TPRN* | *DFNB79* |  |  |  |  | NM_001128228.2 | 4 |
| *TRIOBP* | *DFNB28* |  |  |  |  | NM_001039141.2 | 21 |
| *TSPEAR* | *DFNB98* |  |  |  |  | NM_144991.2 | 12 |
| *USH1C* | *DFNB18* |  |  | *USH1C* |  | NM_153676.3 | 27 |
| *USH1G* |  |  |  | *USH1G* |  | NM_173477.2 | 3 |
| *USH2A* |  |  |  | *USH2A* |  | NM_206933.2 | 71 |
| *WFS1* |  | *DFNA6* |  |  |  | NM_006005.3 | 7 |
| *WHRN* | *DFNB31* |  |  | *USH2D* |  | NM_015404.3 | 12 |
|  |  |  |  |  |  | Σ coding exons | 1914 |
